# The marine natural product mimic MPM-1 is cytolytic and induces DAMP release from human cancer cell lines

**DOI:** 10.1038/s41598-022-19597-4

**Published:** 2022-09-16

**Authors:** Susannah von Hofsten, Marianne Hagensen Paulsen, Synnøve Norvoll Magnussen, Dominik Ausbacher, Mathias Kranz, Annette Bayer, Morten B. Strøm, Gerd Berge

**Affiliations:** 1 Department of Medical Biology, Faculty of Health Sciences, UiT The Arctic University of Norway, 9037 Tromsø, Norway; 2 Department of Pharmacy, Faculty of Health Sciences, UiT The Arctic University of Norway, 9037 Tromsø, Norway; 3 PET Imaging Center Tromsø, University Hospital of North Norway, 9019 Tromsø, Norway; 4 Department of Chemistry, UiT The Arctic University of Norway, 9037 Tromsø, Norway

**Keywords:** Biochemistry, Cancer, Cell biology, Drug discovery, Immunology, Chemistry

## Abstract

Bioprospecting contributes to the discovery of new molecules with anticancer properties. Compounds with cytolytic activity and the ability to induce immunogenic cell death can be administered as intratumoral injections with the aim to activate anti-tumor immune responses by causing the release of tumor antigens as well as damage-associated molecular patterns (DAMPs) from dying cancer cells. In the present study, we report the cytolytic and DAMP-releasing effects of a new natural product mimic termed MPM-1 that was inspired by the marine *Eusynstyelamides*. We found that MPM-1 rapidly killed cancer cells in vitro by inducing a necrosis-like death, which was accompanied by lysosomal swelling and perturbation of autophagy in HSC-3 (human oral squamous cell carcinoma) cells. MPM-1 also induced release of the DAMPs adenosine triphosphate (ATP) and high mobility group box 1 (HMGB1) from Ramos (B-cell lymphoma) and HSC-3 cells, as well as cell surface expression of calreticulin in HSC-3 cells. This indicates that MPM-1 has the ability to induce immunogenic cell death, further suggesting that it may have potential as a novel anticancer compound.

## Introduction

Cancer remains one of the leading causes of death worldwide despite major advances within the field of cancer treatment during recent years^1^. In addition to drug resistance and severe side effects, intratumoral heterogeneity is becoming recognized as a major obstacle for development of novel effective therapies. In this context, cytolytic therapies inducing immunogenic cell death, a specific type of cell death that activates immune responses, are rising as promising new therapeutic tools^2^. Treatment with cytolytic compounds is a novel and attractive option since these can be administered intratumorally, minimizing the effect on healthy cells, while causing the release of tumor antigens from dying cancer cells. Cytolytic compounds that induce immunogenic cell death cause release of damage-associated molecular patterns (DAMPs) into the tumor microenvironment, triggering an immune response. Specifically, surface expression of calreticulin, release of ATP and release of high mobility group box 1 (HMGB1) are generally considered the hallmarks of immunogenic cell death^3^. These DAMPs have recruiting and activating effects on cells of the innate immune system, which can recognize the released tumor antigens. Among these are dendritic cells and macrophages, which upon activation can initiate an adaptive immune response, ultimately leading to the activation of cytotoxic CD8^+^ T cells that recognize and kill cancer cells.

Immunogenic cell death can be induced by several different types of compounds and other cell stressors, which may have different cellular targets. This includes for example conventional DNA-binding agents such as doxorubicin and mitoxantrone^4,5^, some targeted anti-cancer therapies^6^, therapeutic oncolytic viruses^7^, and physical stressors such as ionizing radiation^8^. We have previously shown that the cytolytic peptides LTX-302 and LTX-315 are able to activate adaptive anti-tumor immune responses in mice^9,10^. We have also reported on the cytolytic activity of the ultra-short peptidomimetic LTX-401 (initially reported as BAA-1)^11^, which was recently shown to induce immunogenic cell death in vivo^12,13^. In the present study, we have investigated the cytolytic and immunogenic effects of the novel marine natural product mimic MPM-1.

**Figure 1:**
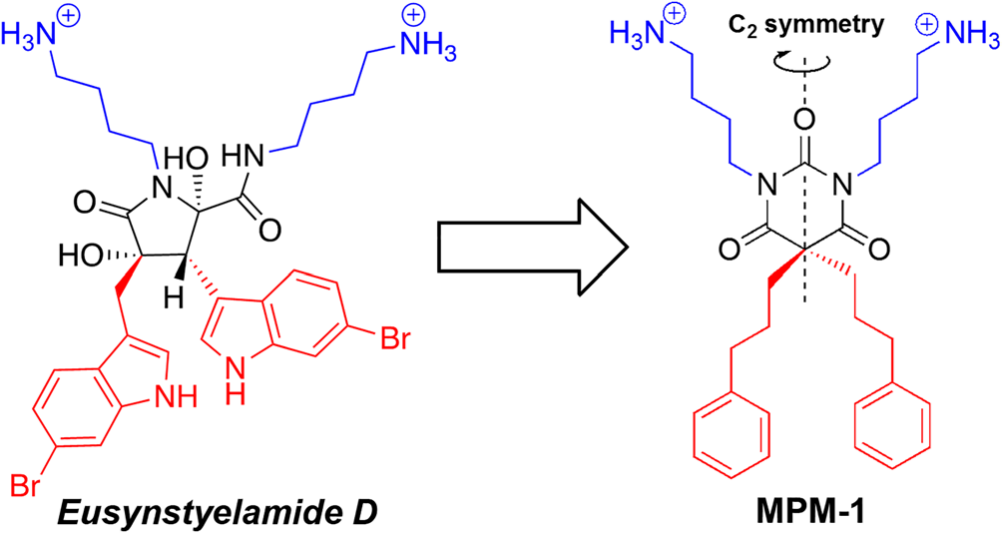
Molecular structures of the marine antimicrobial *Eusynstyelamide D* and the novel marine natural product mimic MPM-1. Cationic groups are colored blue and lipophilic groups are colored red to highlight the amphipathic arrangement in both molecules.

MPM-1 is a synthetic and simplified mimic of a group of marine bioactive compounds referred to as the *eusynstyelamides,* which have previously been isolated from arctic bryozoans (Fig. 1)^14^. The *eusynstyelamides* mainly show antimicrobial activity, and fulfill the minimum pharmacophore model of small cationic antimicrobial peptides by having an amphipathic structure consisting of two cationic and two lipophilic/bulky groups^15^. However, the *eusynstyelamides* are challenging to synthesize due to the complex five-membered dihydroxybutyrolactam ring. Recently, we have reported a class of simplified mimics of the *eusynstyelamides*, where the complex dihydroxybutyrolactam ring is replaced by a barbiturate scaffold^16,17^. This scaffold is rigid and can easily be modified with different cationic and lipophilic groups. Moreover, the barbiturate scaffold has C_2_ symmetry and no stereogenic centers, which implies that synthesis of these compounds will not produce any unwanted stereoisomers of the intended compound. A library of barbiturate *eusynstyelamide* mimics including MPM-1 was created, originally intended as antimicrobial agents. However, during pilot screening experiments, MPM-1 stood out from the other compounds by being able to kill selected cancer cell lines efficiently while showing negligible antimicrobial activity (Bayer and Strøm, unpublished results).

The aim of the present study was to perform an expanded screening of the anti-cancer effect of MPM-1 in addition to studying its mechanism-of-action and ability to induce DAMP release. This represents the first step in elucidating the clinical potential of MPM-1 as a cytolytic compound intended for intratumoral immunotherapy.

## Results

### Synthesis of MPM-1

**Figure 2:**
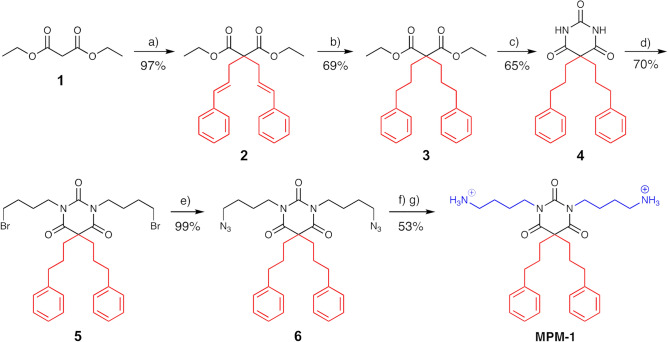
Synthesis of the amphipathic barbiturate MPM-1. Conditions: (**a**) 3-bromo-1-phenyl-1-propene, NaH, DMF, r.t.; (**b**) Pd/C, H_2_ (10 bar), r.t.; (**c**) 10 equiv. urea (dry), NaH, DMF (dry), r.t.; (**d**) 10 equiv. 1,4-dibromobutane, 4 equiv. K_2_CO_3_, DMF (dry), r.t., 18–48 h; (**e**) 3 equiv. NaN_3_, DMF (dry); (**f**) i. NaBH_4_, 1,3-propanedithiol, THF:isopropanol 1:1, r.t.; ii. Boc_2_O, r.t.; (**g**) TFA, CH_2_Cl_2_.

MPM-1 was synthesized based on a procedure previously developed by our group, with some modifications (Fig. 2)^16^. The overall strategy for synthesis of MPM-1 involved dialkylation of malonate ester **1** to attach the lipophilic side chains (giving **2** or **3** as described below), reaction with urea to form the barbiturate scaffold (**4**), *N*-alkylation of the barbiturate (**5**), followed by azide conversion (**6**), and final reduction of the azide groups to give MPM-1.

Two different alkylation reagents were tested to give the dialkylated malonate ester **3**; 1-bromo-3-phenylpropane and 3-bromo-1-phenyl-1-propene. Dialkylation of the malonate ester **1** with 1-bromo-3-phenylpropane gave a mixture of starting material, mono and dialkylated malonate ester (**3**), which proved difficult to separate using flash chromatography. Both the weak base potassium carbonate (K_2_CO_3_) and the stronger base sodium hydride (NaH) were tested as a base in the reaction. Neither of the two bases gave satisfying amounts of the dialkylated malonate ester **3,** so an alternative route was explored. In the second route we used 3-bromo-1-phenyl-1-propene as the alkylating agent, which gave the dialkylated malonate ester **2** in high yield (97%) but involved an additional hydrogenation step to achieve **3** (67% overall yield). The dialkylated malonate ester (**3**) was further condensed with urea to give barbiturate **4** (65% yield). *N*,*N*-Dialkylation of **4** using an excess of 1,4-dibromobutane under basic conditions gave **5** in 70% yield. The *N*,*N*′-dialkylated barbiturate **5** was converted to the corresponding azide derivative **6** using sodium azide (NaN_3_) (99% yield). Reduction of the azide groups in **6** with a catalytic amount of propane-1,3-dithiol and sodium borohydride (NaBH_4_), and subsequent Boc-protection with Boc_2_O and Boc deprotection with 2,2,2-trifluoroacetic acid (TFA) gave the target molecule MPM-1 in 53% yield. Of note, the Boc-protection and Boc-deprotection steps were necessary in order to ease purification by flash chromatography and increasing the purity of MPM-1. NMR data for the synthesis can be found as Supplementary Data S1.

### MPM-1 is cytotoxic to a large selection of cancer cell lines

The cytotoxic effect of MPM-1 was assessed by determining its IC50 value for a panel of cell lines (Table 1 and Supplementary Fig. S1). The panel represented a selection of cancerous, non-cancerous, adherent, non-adherent, and multidrug resistant cells. As a preliminary measurement of drug toxicity, the hemolytic activity of MPM-1 was also measured. MPM-1 had no hemolytic activity against human red blood cells (IC50 > 500 μM), but effectively killed all other cell types tested. The obtained IC50 values were of similar magnitude, ranging from 4.13 μM for PBMCs to 18.54 μM for MRC-5. Apart from the lack of hemolysis of human red blood cells, there was no trend towards selectivity for cancerous over non-cancerous cell lines. For this reason, our subsequent studies focused mainly on the oral cancer cell line HSC-3, which represents a solid tumor. However, the mean IC50 value for suspension cells was significantly lower than for adherent cell lines (6.09 ± 1.76 vs. 14.00 ± 3.12, p = 0.002). Since this could be indicative of MPM-1’s mode of action, one adherent (HSC-3) and one suspension (Ramos) cell line was included in several of the mechanistic studies. In these studies, the cells were treated with concentrations of MPM-1 equal to their respective IC50 values or multiples thereof.

**Table 1:** MPM-1 IC50 values obtained from MTS cytotoxicity assays after four hours of incubation. The values represent the mean from three independent experiments ± standard deviation.

Cell line	Type^a^	Site of origin	IC50 (µM) ± SD
A375	A	Human melanoma	14.52 ± 0.22
HepG2	A	Human hepatocellular carcinoma	17.26 ± 2.50
HSC-3	A	Human oral squamous cell carcinoma	8.53 ± 0.57
HT-29	A	Human colorectal adenocarcinoma	15.68 ± 0.33
SK-N-AS	A	Human neuroblastoma	15.94 ± 0.23
MCF-7	A	Human breast adenocarcinoma (multidrug resistant)	14.06 ± 2.71
Jurkat	S	Human T cell leukemia	6.62 ± 1.60
Ramos	S	Human B cell lymphoma	7.53 ± 2.01
B16F1	A	Murine melanoma	13.72 ± 0.61
GL261-Luc2	A	Murine glioblastoma	11.04 ± 2.88
MRC-5	A	Non-cancerous human lung fibroblast	18.54 ± 2.68
HUVEC	A	Non-cancerous human umbilical endothelium	10.73 ± 3.63
PBMC	S	Human peripheral blood mononuclear cells	4.13 ± 0.31

^a^A = Adherent cells, S = Suspension cells.

### The cytotoxicity of MPM-1 is dependent on compound concentration and cell density

**Figure 3:**
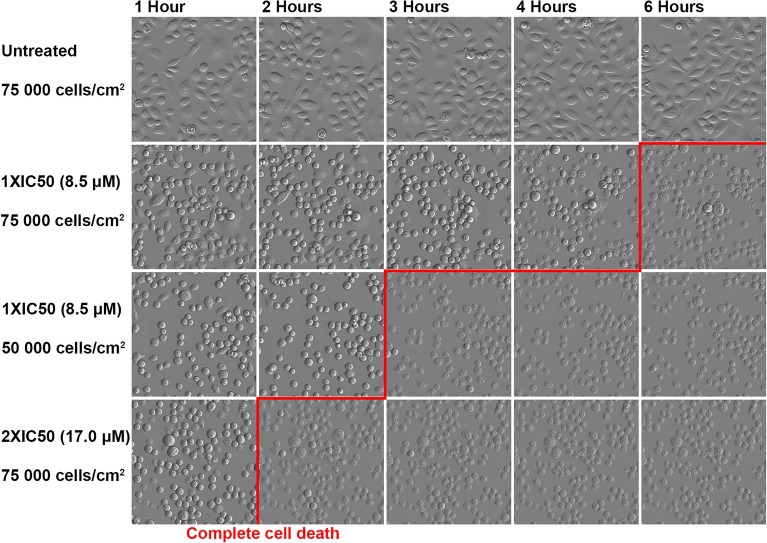
The rate at which MPM-1 kills cells is dependent on concentration and cell density. HSC-3 cells were seeded at different densities, treated with 1xIC50 or 2xIC50 MPM-1, and subsequently imaged continuously.

To study the effect of treating cells with MPM-1 in different concentrations as well as with different cell densities, live cell imaging of HSC-3 cells treated with MPM-1 was performed. It was found that the density of cells seeded for experiments with MPM-1 greatly affected the cytotoxicity of MPM-1. At approximately 80% confluence (7.5 × 10^4^ cells/cm^2^), HSC-3 cells appeared to be dead after six hours when treated with 1xIC50 MPM-1 (Fig. 3). This could be seen from the cells’ changed morphology. They became rounded, stopped moving and by 6 h looked completely damaged (Supplementary Video S1). When decreasing the number of cells per well to 5 × 10^4^ cells/cm^2^, cell death appeared after only three hours with the same concentration of MPM-1. Increasing the concentration of MPM-1 to 2xIC50, but keeping the original number of cells per well (7.5 × 10^4^ cells/cm^2^), also greatly accelerated the rate of cell death, which occurred after only two hours. In contrast, when decreasing the concentration of MPM-1 to ½xIC50, reaching complete cell death took approximately 15 h (Supplementary Video S2). This indicates that the cytotoxicity of MPM-1 is directly related to the number of available molecules per cell.

### MPM-1 causes vacuolization and necrosis

**Figure 4:**
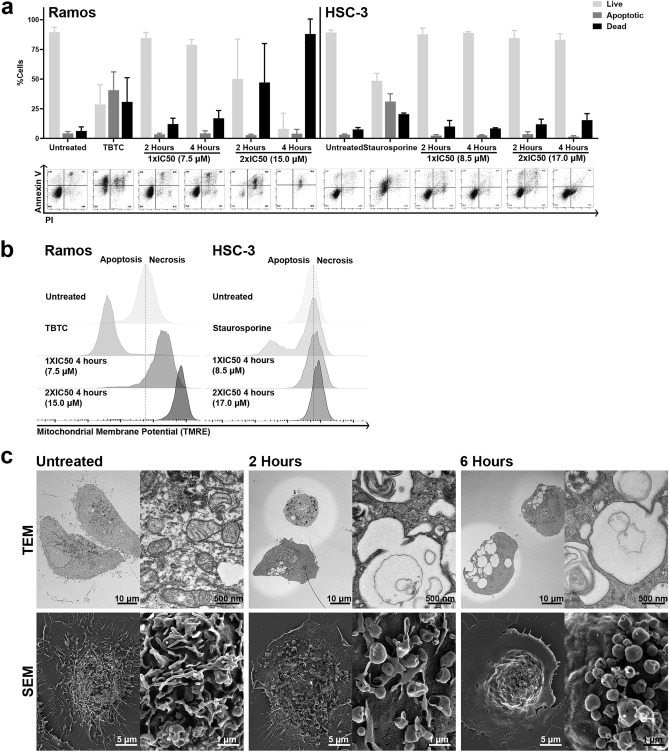
MPM-1 causes necrosis and vacuolization. (**a**) Ramos and HSC-3 cells were analyzed by flow cytometry for cell surface exposure of Annexin V. Cells were treated with 1xIC50 or 2xIC50 MPM-1 for 2 or 4 h. TBTC (2 µM, 2 h) and Staurosporine (100 nM, 24 h) were used as apoptosis controls for Ramos and HSC-3 cells, respectively. The graph shows the percentage of live (PI^–^/Annexin V^–^), apoptotic (PI^–^/Annexin V^+^), and dead (PI^+^) cells for each condition, determined from three independent experiments, with error bars representing the standard deviation. (**b**) The mitochondrial membrane potential in Ramos and HSC-3 cells treated with 1xIC50 or 2xIC50 MPM-1 for four hours was assessed by flow cytometric analysis of TMRE fluorescence. The graph illustrates the results from three independent experiments. (**c**) Transmission (TEM) and scanning (SEM) electron microscopy images of untreated HSC-3 cells and HSC-3 cells treated with 1xIC50 MPM-1 for 2 or 6 h.

To study the mode of death induced by MPM-1, HSC-3 and Ramos cells were analyzed for the cell surface exposure of phosphatidylserine (PS), which characterizes apoptosis. This can be detected by staining with fluorescently labeled Annexin V, which binds to PS, and propidium iodide (PI), which only penetrates cells with ruptured cell membranes. Treatment of HSC-3 and Ramos cells with known inducers of apoptosis (Staurosporine and TBTC) resulted in the appearance of a large annexin V^+^/PI^−^ apoptotic population, whereas treatment of cells with MPM-1 did not result in the formation of such a population (Fig. 4a). However, the percentage of PI^+^ events did increase, indicating that cells had died, but not from apoptosis.

The mitochondrial membrane potential in cells treated with MPM-1 was also analyzed by flow cytometry, using the fluorescent dye tetramethylrhodamine (TMRE) (Fig. 4b). In healthy cells, the mitochondrial intermembrane space is positively charged, while the mitochondrial matrix is negatively charged. This proton gradient creates a potential, which is referred to as the mitochondrial membrane potential. TMRE is positively charged and therefore accumulates in the mitochondrial matrix^18^. An early sign of apoptosis is a collapse of the mitochondrial membrane potential, also referred to as a depolarized mitochondrial membrane potential, which causes the mitochondrial matrix to be less negatively charged. This in turn causes less TMRE to be sequestered by the mitochondria, which could be observed when Ramos and HSC-3 cells were treated with their respective apoptosis controls (Fig. 4b). When cells were treated with MPM-1, the TMRE fluorescence increased instead, indicating that the mitochondrial matrix had become more negatively charged, i.e. that the mitochondrial membrane potential was hyperpolarized. This effect was especially prominent in Ramos cells, but a slight increase in fluorescence could be seen for HSC-3 cells as well. Hyperpolarization of the mitochondrial membrane potential is typical for necrotic cells^19^. Taken together, the flow cytometric analyses indicate that MPM-1 causes cells to undergo a form of necrosis.

To further study the morphological changes induced in HSC-3 cells treated with MPM-1, scanning and transmission electron microscopy (SEM/TEM) images were acquired (Fig. 4c). No typical signs of apoptosis, such as chromatin condensation or distorted mitochondria, could be observed upon treatment with MPM-1. Both nuclei and mitochondria were unaffected, indicating a necrotic mode of cell death. Instead, SEM images revealed major structural changes on the cell membrane of HSC-3 cells. Untreated HSC-3 cells had a rough surface covered with microvillus-like protrusions. Upon treatment with MPM-1, these protrusions generally disappeared, rendering the membrane surface smoother. Simultaneously, formation of vesicles on the surface of the cell membrane could be observed. TEM images demonstrated the formation of large intracellular single-membraned vesicles or vacuoles.

### Effect of MPM-1 on autophagy and lysosomes

Increased autophagy has been suggested as a survival mechanism for drug-treated cells, and it has also been coupled to vacuolization of dying cells^20^. Autophagy is a process that cells use to degrade and reuse cellular content. A double-membraned autophagosome forms around the content that should be degraded and subsequently fuses with a lysosome, creating a single-membraned autolysosome where lysosomal enzymes degrade the content^21^. We hypothesized that the large vesicles observed in Fig. 4c upon treatment of HSC-3 cells with MPM-1 might be coupled to autophagy or other lysosomal degradation pathways.

**Figure 5:**
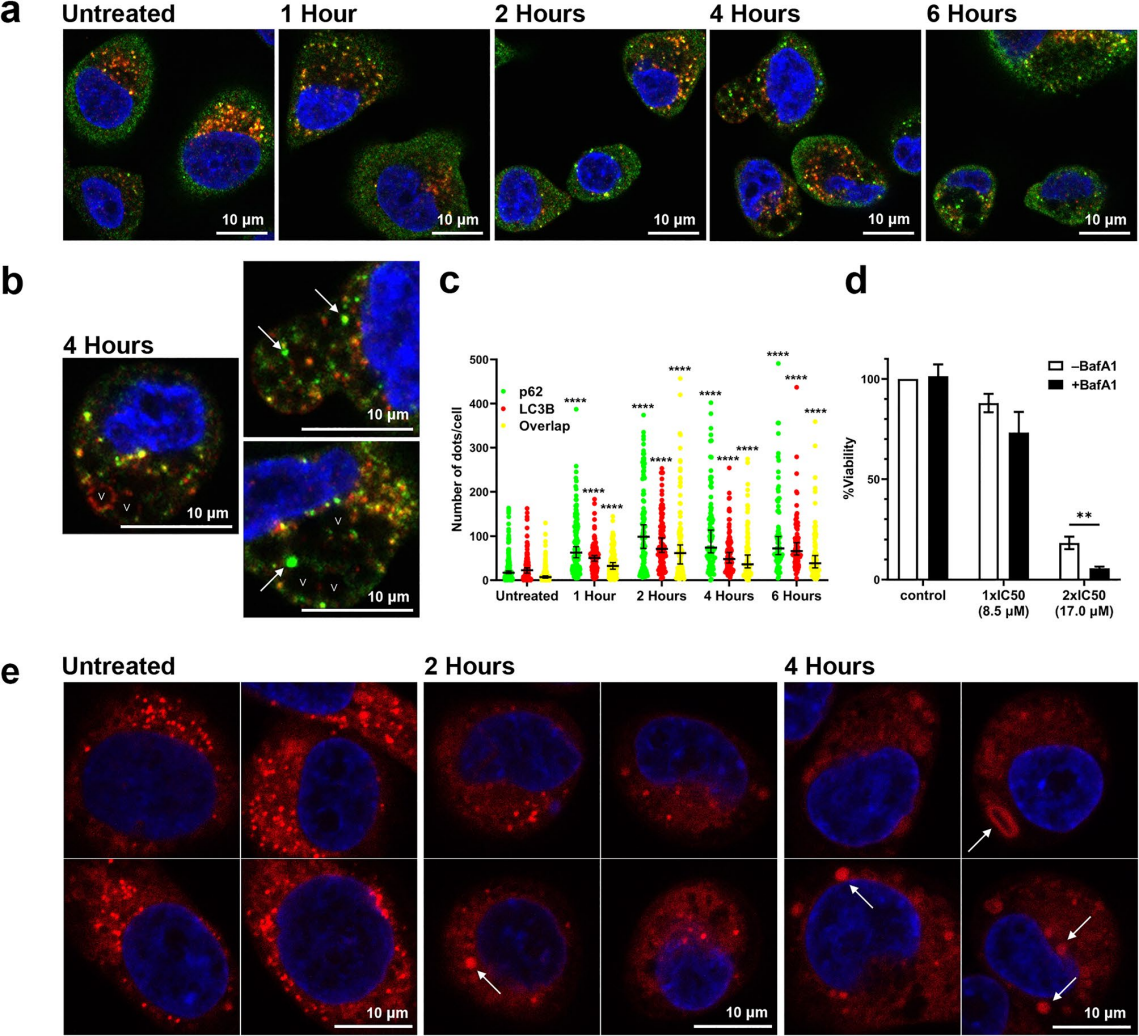
MPM-1 causes perturbation of autophagy and lysosomal swelling. (**a**) Immunofluorescence microscopy images of untreated HSC-3 cells and HSC-3 cells treated with 1xIC50 MPM-1 for 1, 2, 4 or 6 h before being stained for presence of p62 (green) and LC3B (red). Examples of vesicles (V) and p62 aggregates (arrows) are shown in images taken after 4 h of treatment (**b**). The number of p62/LC3B/overlapping dots per cell was counted in > 80 cells for each condition and the results are visualized in (**c**). Horizontal lines denote the median and error bars show 95% CI. Significant differences between treated and untreated groups were determined by separate Kruskal–Wallis tests with Dunn’s post-hoc for the p62, LC3B, and overlap data. (**d**) Viability of HSC-3 cells after treatment with 1xIC50 or 2xIC50 MPM-1 in the presence or absence of bafilomycin A1 (BafA1) (100 nM) was determined by the MTS assay. Bars represent the mean from three independent experiments with error bars denoting the standard deviation. Significant differences were determined by unpaired t-test (**e**) Immunofluorescence microscopy images of untreated HSC-3 cells and HSC-3 cells treated with 1xIC50 MPM-1 for 2 or 4 h before being stained with Lysotracker Deep Red. Arrows indicate enlarged lysosomes.

MPM-1 treated HSC-3 cells were stained for the autophagy markers p62 (green) and LC3B (red), and immunofluorescence confocal microscopy images were acquired. Normally, LC3B coats the membrane of autophagosomes, while p62 is involved in sequestering the content to be degraded. Co-localization of p62 and LC3B (yellow) can therefore be used as a marker of autophagosomes^22^. The staining with p62 and LC3B revealed the presence of the same large vesicles as were seen in the electron microscopy images, here seen as empty black circles (Fig. 5a). Overall, it was not possible to determine whether the vesicles were specifically connected to the presence of LC3B or p62. With a few exceptions, the large vesicles were not coated by LC3B or p62, indicating that they were not autophagosomes. It is however worth mentioning that a small selection of vesicles were clearly coated by LC3B, as can be seen in one of the images acquired after four hours of treatment (Fig. 5b). A number of smaller autophagosomes (yellow dots) could be seen in both untreated and MPM-1 treated cells. Quantification of the number of co-localized p62 and LC3B dots per cell revealed that MPM-1 treated cells on average contained a higher number of autophagosomes (Fig. 5c). There was also a significant increase in the total number of only green (p62) or only red (LC3B) dots in MPM-1 treated cells. The appearance of large, green dots was especially prominent, and revealed that aggregates of p62 had been formed. Since p62 is a substrate of autophagy, the total amount of it is expected to decrease in cells where the autophagic activity (autophagic flux) is high. Accumulation of p62 and of autophagosomes instead often occurs when autophagy is inhibited, indicating that this might be an effect of MPM-1^22^.

Since autophagy related protein 7 (ATG7) knockout HeLa cells were readily available to us, we used these to study whether the process of autophagy could affect the sensitivity of cells towards MPM-1. The ATG7 KO cells are not able to perform autophagy because ATG7 is essential for the formation of autophagosomes^21^. The IC50-value of MPM-1 was determined for wild type (WT) HeLa cells and the ATG7 knockout HeLa cells. There was no significant difference between the IC50-value obtained for WT and ATG7 KO HeLa cells (24.30 ± 1.73 µg/ml vs. 22.97 ± 0.35 µg/ml, p = 0.3) (Supplementary Fig. S2), indicating that autophagy could not protect the HeLa cells from MPM-1.

However, we further studied the role of autophagy by treating HSC-3 cells with MPM-1 in the presence of the late-stage autophagy inhibitor bafilomycin A1 to see if this would affect the cytotoxicity of MPM-1. Bafilomycin A1 is an inhibitor of the V-ATPase which is present on lysosomes and keeps their internal pH low. Bafilomycin A1 therefore causes the lysosomal pH to increase, in turn causing lysosomal dysfunction and inhibition of fusion between autophagosomes and lysosomes^23^. Bafilomycin A1 alone was not cytotoxic during the time span of the assay, but the results revealed that cells were more sensitive to MPM-1 in the presence of bafilomycin A1 than in its absence (Fig. 5d). The same tendency was observed for both WT and ATG7 KO HeLa cells as well (Supplementary Fig. S3).

To study the effect of MPM-1 on lysosomes, HSC-3 cells were stained with the fluorescent dye Lysotracker Deep Red, which stains lysosomes and other acidic cellular compartments, and confocal microscopy images were acquired (Fig. 5e). Untreated cells contained a high number of small and acidic lysosomes, as seen by the bright lysotracker signal. In cells treated with MPM-1 the distribution of the lysotracker dye was more diffuse and less intense, making it difficult to distinguish individual lysosomes from each other. Thus, it was not possible to perform automatic identification of lysosomes and quantification of size and lysotracker intensity. However, it was evident that MPM-1 influenced lysosomal morphology. Several of the MPM-1 treated cells contained lysosomes that were visibly enlarged as compared to the lysosomes in untreated cells. The enlarged lysosomes generally seemed to have a relatively weak lysotracker signal, indicating that their internal pH was higher than the pH of lysosomes in untreated cells. Taken together, these results reveal that MPM-1 has an effect on lysosomes, which in turn may be the reason for the observed accumulation of autophagosomes and p62.

### MPM-1 induces release and exposure of DAMPs

**Figure 6:**
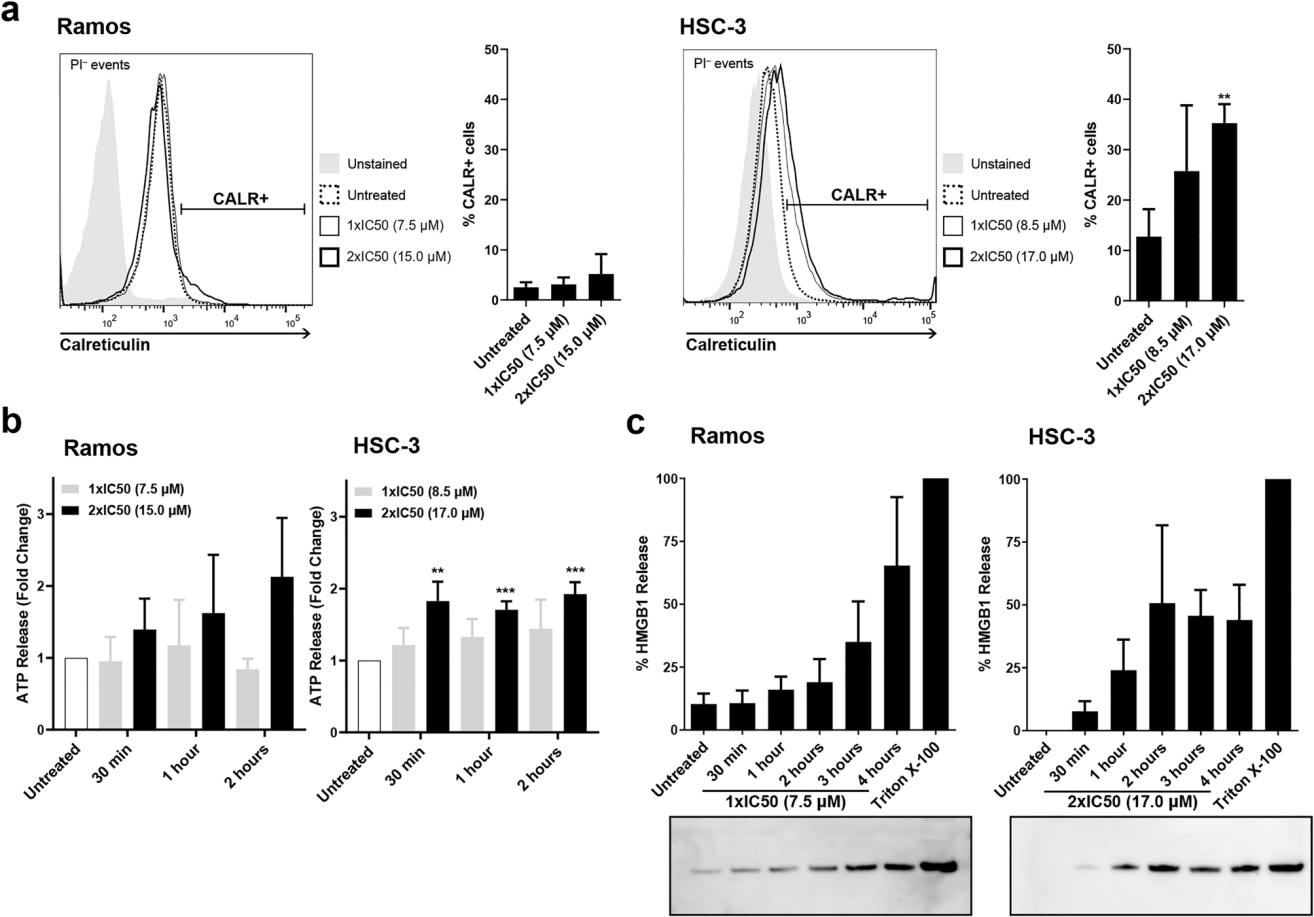
MPM-1 induces the release and exposure of DAMPs related to immunogenic cell death. (**a**) Cell surface exposure of calreticulin by Ramos and HSC-3 cells treated with 1xIC50 or 2xIC50 MPM-1 for four hours was assessed by flow cytometry. Calreticulin expressing cells were identified among live (PI negative) cells. The bar graph shows the mean percentage of calreticulin expressing cells from three separate experiments with error bars denoting the standard deviation. Significant differences between treated and untreated groups were determined by unequal variances t-test. (**b**) Release of ATP from Ramos and HSC-3 cells treated with MPM-1 was assessed by a firefly luminescence assay. Bars represent the mean ATP release from five individual experiments, expressed as fold increase relative to untreated (control) cells. Significant differences between treated and untreated groups were determined by repeated measures ANOVA and Dunnett’s post-hoc. (**c**) Release of high mobility group box 1 (HMGB1) from cells treated with MPM-1 was assessed by Western blotting. Untreated and Triton X-100 treated cells served as negative and positive control, respectively. Bars represent the mean from three independent experiments with error bars denoting the standard deviation. The blots have been cropped for presentation in this figure. Original blots are presented in Supplementary Fig. S4.

To study the immunogenic potential of MPM-1, we tested whether the cell death induced by MPM-1 caused release and exposure of DAMPs specifically related to immunogenic cell death. Flow cytometry was used to detect cell surface calreticulin in Ramos and HSC-3 cells treated with MPM-1. For Ramos cells, only a very small and statistically insignificant increase in cell surface calreticulin was observed after treatment with MPM-1. However, HSC-3 cells treated with 2xIC50 MPM-1 for four hours showed a significantly increased amount of cell surface calreticulin, as compared to untreated cells (Fig. 6a). Release of ATP into the cell media by cells treated with MPM-1 was analyzed with a firefly luminescence-based assay. For Ramos cells treated with MPM-1, only a small amount of ATP could be detected in the supernatant. The same was observed for HSC-3 cells treated with 1xIC50 MPM-1, but with 2xIC50 MPM-1, the ATP levels increased significantly (Fig. 6b). Release of HMGB1 was analyzed by Western blot and demonstrated that for Ramos cells, HMGB1 release occurred rapidly upon treatment with MPM-1 (Fig. 6c). The supernatant of untreated Ramos cells already contained detectable levels of HMGB1, but upon treatment with 1xIC50 MPM-1, the amount of HMGB1 in the supernatant gradually increased with time. For HSC-3 cells, no HMGB1 was detectable in the supernatant of untreated cells, but upon treatment with 2xIC50 MPM-1, HMGB1 was released. In summary, these results demonstrate that MPM-1 does induce the release and exposure of DAMPs related to immunogenic cell death.

## Discussion

Our results demonstrate that the newly developed marine natural product mimic MPM-1 may have potential as an intratumoral immunotherapy. MPM-1 is clearly cytolytic and rapidly induced cell death in all cell lines tested. This included the multi-drug resistant breast adenocarcinoma cell line MCF-7. This non-selective killing of cells indicates that MPM-1 likely targets a site of action that is present in most cells. Inevitably, this means that also non-malignant cells can be killed by MPM-1 in an in vivo setting. However, the ability to target, and thereby release antigens from, any cell in a heterogenic tumor is the main goal of cancer treatment with cytolytic compounds. Moreover, the intended administration route for MPM-1 is as an intratumoral injection, which can help limit the amount of damage to non-malignant healthy cells.

It is worth emphasizing that human red blood cells were not affected by treatment with MPM-1. This finding suggests that MPM-1’s primary target is an intracellular structure. Unlike most other cells, red blood cells do not possess organelles or a nucleus. The electron microscopy images of HSC-3 cells treated with MPM-1 showed that large intracellular vesicles had been formed and suggests that MPM-1 affects some part of vesicular transport or degradation. MPM-1 is a small, weakly basic and amphipathic compound. This means that it fits the description of *lysosomotropic compounds*, which induce cell death by causing lysosomal dysfunction^24,25^. Lysosomotropic compounds are also known not to lyse red blood cells, since they do not possess lysosomes. Lysosomotropic compounds have lipophilic features that allow them to cross through the lipid cell membrane in their neutral (deprotonated) form, while their basic nature leads them to accumulate within the acidic lysosomes. Due to the basic features of MPM-1, the majority of molecules are expected to be fully protonated at physiological pH and not able to cross a lipophilic cell membrane. However, due to the equilibrium between protonated and neutral molecules, a small fraction of neutral molecules will be present and likely able to diffuse into cells and their organelles. The acidic environment inside the lysosomes will then cause MPM-1 to become protonated and thereby trapped within the lysosomes. This is the accepted mechanism of action for lysosomotropic compounds^24^. As more and more molecules become trapped, osmotic pressure causes water to diffuse into the lysosomes, which then adopt the appearance of large vacuoles. The influx of water into lysosomes makes the lysosomal pH higher and causes biological dysfunction of the lysosomes. Additionally, the trapped molecules can function as detergents, causing disruption of the lysosomal membrane, leakage of lysosomal enzymes, and subsequent cell death^24^.

The imaging results obtained in the present study, which demonstrated the appearance of large empty vacuoles in HSC-3 cells treated with MPM-1, support the idea that MPM-1 is a lysosomotropic compound. Moreover, since lysosomes play a central role in autophagy, their dysfunction is expected to disturb the autophagic flux. Again, this fits well with the observed results for MPM-1. Accumulation of autophagosomes, as well as LC3B and p62 aggregates is a well-known effect of disrupted fusion of autophagosomes with lysosomes^25^. Interestingly, a few of the large vesicles were clearly coated by LC3B, suggesting that they were of autophagic origin. A possibility is that the LC3B coated vesicles were autolysosomes, which are also acidic and therefore might trap MPM-1 in the same way as other lysosomes. This also fits the electron imaging results, which showed that the large vesicles had single membranes.

For some lysosomotropic compounds, including the well-studied antimalarial drug chloroquine, their main mechanism of inducing cell death is thought to be via their accumulation in lysosomes^25^. It has been demonstrated that bafilomycin A1 can protect cells from chloroquine induced cell death, an effect which may partially be due to decreased sequestration of chloroquine in lysosomes when the lysosomal pH is increased^27^. However, many well-known drugs and cytotoxic compounds are lysosomotropic even though their main target is located in other parts of the cell. For such compounds, increased lysosomal pH causes less compound to be sequestered in lysosomes and more to reach the main target. One example is the DNA intercalating agent doxorubicin, which has been shown to accumulate in lysosomes^28^. Upon treatment with bafilomycin A1 to increase the lysosomal pH, it was demonstrated that doxorubicin re-located from the lysosomes to a more diffuse distribution in the whole cell and subsequently induced more cell death^28^. Similarly, when HSC-3 cells were treated with bafilomycin A1, they became more sensitive to treatment with MPM-1. Since bafilomycin A1 itself was not cytotoxic, this indicates that the effect was synergistic. Furthermore, this result suggests that the main target for MPM-1 may not be lysosomes but possibly another organelle or structure located in the cytoplasm or nucleus.

We hypothesized that autophagy could affect the sensitivity of cells towards MPM-1. The fact that inhibition of autophagy with bafilomycin A1 caused increased cell death by MPM-1 supports this hypothesis. However, the results obtained with the HeLa cells, which showed that there was no difference in sensitivity towards MPM-1 between WT and autophagy deficient ATG7 KO HeLa cells, does not. Despite the fact that treatment with bafilomycin A1 and knockout of ATG7 both cause inhibition of autophagy, it is important to note that they do so through different mechanisms. Bafilomycin A1 indirectly inhibits autophagy by causing lysosomal dysfunction which subsequently inhibits the fusion of autophagosomes with lysosomes. Knockout of ATG7 directly inhibits autophagy by inhibiting the formation of autophagosomes without affecting lysosomes. These results thus suggest that it is the dysfunction of lysosomes which sensitizes cells towards MPM-1 rather than the inhibition of autophagy per se. Consequently, the ATG7 KO HeLa cells were equally sensitive to MPM-1 as WT HeLa cells. The same phenomenon is observed for WT and ATG7 silenced cells treated with chloroquine^26^. Nevertheless, autophagy is a complex process, and it cannot be ruled out that it may play some role in cells’ response to treatment with MPM-1 or that the disturbance of the autophagic flux seen upon treatment of HSC-3 cells with MPM-1 may have implications for its potential as an anticancer compound.

Autophagy is considered an instrumental cellular process for most cancer cells. For this reason, lysosomotropic compounds such as chloroquine and hydroxychloroquine have been tested as anti-cancer agents in several studies and clinical trials, with some showing promising results^25^. Chloroquine is usually given as a systemic treatment, which means that it can affect many different cell types in the body. The intratumoral injection route suggested for MPM-1 thus represents an alternative treatment mode for lysosomotropic compounds, which could be used to target cancer cells more specifically.

The live cell imaging of HSC-3 cells indicated that the potency of MPM-1 was not directly related to concentration of the compound per se, but rather to the exact number of molecules available per cell. This was demonstrated by the fact that it took longer to reach cell death when the cell density was higher, and that low concentrations of MPM-1 needed more time to cause cell death as compared to high concentrations. This phenomenon is often referred to as the inoculum effect, and it has been demonstrated to apply for several types of compounds, including doxorubicin^29^. The fact that the suspension cell lines had significantly lower IC50 values compared to adherent cells may be due to the lack of adherence to a surface, making their entire cell membrane available for penetration by MPM-1, possibly resulting in a high intracellular drug concentration being reached sooner. This phenomenon could be relevant for several types of compounds, indicating that when planning drug screening projects, the types of cell lines that are included and compared with each other should be carefully considered.

Many chemotherapeutic and lysosomotropic agents cause cell death by inducing apoptosis^4,30^. However, MPM-1 did not induce any of the signs that are typically associated with apoptosis, such as exposure of phosphatidylserine, chromatin condensation or depolarized mitochondrial membrane potential. Instead, the death induced by MPM-1 was accompanied by morphological and biochemical features typical of necrosis. Historically, necrosis has been regarded as an accidental form of cell death, but the number of recognized modes of cell death has greatly expanded over the last few decades and now includes regulated forms of necrosis as well^31^. Inducing regulated forms of necrosis has been suggested as a potentially more beneficial approach to cancer therapy than inducing immunogenic apoptosis^32^. The reason for this being that many tumors have developed resistance to apoptosis and therefore do not respond to apoptosis inducing chemotherapies^32^. In addition, apoptotic cells express phosphatidylserine, which is immunosuppressive. Phosphatidylserine promotes removal of apoptotic cells while simultaneously inhibiting unnecessary inflammation. It is also reported that phosphatidylserine plays a role in the tumor microenvironment, where it has immunosuppressive functions on immune cells^33^. Since the goal of intratumoral immunotherapy is to activate immune responses, the fact that MPM-1 does not induce expression of phosphatidylserine is promising.

The exposure of calreticulin on the surface of MPM-1 treated HSC-3 cells supports the idea that the cell death induced by MPM-1 is regulated. HMGB1 and ATP can be released from cells dying of accidental necrosis, but exposure of calreticulin requires regulated signaling and is not typically associated with necrotic cells^34^. The fact that no cell surface calreticulin was detected in MPM-1 treated Ramos cells may indicate that MPM-1 caused a more classical type of necrosis in these cells. However, since Ramos cells do not originate from a solid tumor, they do not represent the type of cancer that would benefit from intratumoral injection with MPM-1. Thus, the finding that MPM-1 could cause translocation of calreticulin to the outside of HSC-3 cells, which do originate from a type of solid tumor, was particularly interesting.

Together with the release of HMGB1 and ATP, the exposure of calreticulin on HSC-3 cells indicates that MPM-1 may have the ability to induce immunogenic cell death. Cell surface calreticulin functions as an “eat me” signal, which is important for the effective phagocytosis of dying cancer cells by cells of the innate immune system and their subsequent cross-presentation of tumor antigens to cells of the adaptive immune system^4^. Exposed calreticulin binds to CD91, mainly expressed by dendritic cells and macrophages^35^. Release of ATP functions as a “find me” signal. By binding to the purinergic receptors P2RY2 and P2RX7 on dendritic cells and macrophages, ATP stimulates their recruitment and activation^36,37^. HMGB1 can bind different receptors, including Toll-like receptor 4 (TLR4) on dendritic cells, which is most relevant for immunogenic cell death. Upon binding to TLR4, HMGB1 promotes antigen processing and cross-presentation of tumor antigens^38^.

A widely accepted notion is that whether a compound truly has the ability to induce immunogenic cell death or not, can only be determined through in vivo experiments^37^. We have previously used a model where tumors are established subcutaneously in immunocompetent mice and then treated by intratumoral injections^10^. Mice with complete tumor regression are then given a secondary challenge with the same tumor cells. Absent or slow tumor growth is interpreted as a sign that the compound used for treatment did induce immunogenic cell death. We are currently performing extensive in vivo studies of this type with MPM-1.

The present study is the first report on the cytolytic and mechanistic effects of MPM-1. We have shown that MPM-1 effectively kills many different cancer cells, while affecting autophagy and causing the release and exposure of DAMPs related to immunogenic cell death. Moreover, the unique marine background of MPM-1 highlights the fact that there is still much unexplored potential in molecules derived from arctic marine species.

## Methods

### Reagents and equipment

MPM-1 (Mw 734.74) was synthesized as described below and dissolved in 10 mM Tris–HCl buffer (pH 7.4) to 1 mg/ml. In all cell-based assays, MPM-1 was further diluted in cell culture medium. Tributylchlorotin (TBTC) was from Sigma-Aldrich (St. Louis, MO, USA), and Staurosporine was from Abcam (Cambridge, UK).

### Synthesis of MPM-1

The synthesis of MPM-1 was similar to that described in our original report on amphipathic barbiturates^16^. All reagents and solvents were purchased from commercial sources and used as supplied. Anhydrous DMF was prepared by storage over 4 Å molecular sieves. The hydrogenation with Pd/C at higher pressure (8–10 bar) were carried out on a Parr Instrument, Series 4590 Micro Stirred reactor, 50 ml, attached to a Parr 4843 Modular Controller. Reactions were monitored by thin-layer chromatography (TLC) with Merck pre-coated silica gel plates (60 F_254_). Visualization was accomplished with either UV light or by immersion in potassium permanganate or phosphomolybdic acid (PMA) followed by light heating with a heating gun. Purifications using normal phase flash chromatography were either done by normal column chromatography using Normalsil 60, 40–63 mm silica gel or by automated normal phase flash chromatography (Heptane/EtOAc) with the sample preloaded on a Samplet^®^ cartridge belonging to a Biotage SP-1. Purification of reactions by reversed phase (RP) C_18_ column chromatography (water with 0.1% TFA/acetonitrile with 0.1% TFA) was also executed on an automated purification module with the sample preloaded on a Samplet^®^ cartridge. The sample used for biological testing were determined to be of > 95% purity. NMR spectra were obtained on a 400 MHz Bruker Avance III HD equipped with a 5 mm SmartProbe BB/^1^H (BB = ^19^F, ^31^P–^15^N). Data are represented as follows: chemical shift, multiplicity (s = singlet, d = doublet, t = triplet, q = quartet, p = pentet, h = heptet, m = multiplet), coupling constant (*J*, Hz) and integration. Chemical shifts (δ) are reported in ppm relative to the residual solvent peak (CDCl_3_: δ_H_ 7.26 and/or 1.56, and δ_C_ 77.16; CD_3_OD: δ_H_ 3.31 and δ_C_ 49.00). Positive and negative ion electrospray ionization mass spectrometry (ESI–MS) was conducted on a Thermo electron LTQ Orbitrap XL spectrometer.

*Diethyl 2,2-dicinnamylmalonate* (**2**). To a stirred solution of diethyl malonate (2.0 g, 1.89 ml, 12.48 mmol) in DMF (20 ml) at 0ºC, NaH (630 mg, 26.22 mmol, 2.1 equiv.) was added slowly. A solution of 3-bromo-1-phenyl-1-propene (5.16 g, 26.22 mmol, 2.1 equiv.) in DMF (25 ml) was then added. The reaction was kept stirring at RT over night. The reaction mixture was diluted with EtOAc (100 ml), water (20 ml) and 10% citric acid (20 ml). The layers were separated and the organic phase was washed with water (4 × 50 ml) and brine. The organic phase was dried over Na_2_SO_4_, filtered and concentrated. The crude product was dissolved in CHCl_3_, and adsorbed on Celite. The product was purified on a silica column using 0–5% EtOAc/pentane as mobile phase to give **2** (4.801 g, 97%) as a white powder. ^1^H NMR (400 MHz, Chloroform-*d*) δ 7.37 – 7.27 (m, 8H), 7.25–7.18 (m, 2H), 6.46 (d, 2H), 6.10 (dt, *J* = 15.5, 7.5 Hz, 2H), 4.22 (q, *J* = 7.1 Hz, 4H), 2.85 (dd, *J* = 7.5, 1.4 Hz, 4H), 1.26 (t, *J* = 7.1 Hz, 6H). HRMS-ESI: C_25_H_28_NaO_4_^+^[M + Na]^+^ calcd: 415.1880, found: 415.1868.

*Diethyl 2,2-bis(3-phenylpropyl)malonate* (**3**). The procedure was performed in a Parr hydrogenation apparatus under pressure (10 bar). Pd/C was weighted out in a test tube, soaked in EtOH (3 ml) and poured into the “bomb”. **1a** (2.5 g, 6.4 mmol) was dissolved in EtOH and added to the “bomb”. The bomb was mounted on the Parr hydrogenation apparatus, evacuated and refilled 6 times with H_2_ and stirred at r.t. for 48 h. After purging, the reaction mixture was filtered through a pad of celite and concentrated. The resulting brown oil was dissolved in 40 ml CHCl_3_ and concentrated to remove remaining EtOH. Adding heptane revealed some Pd/C particles so the solution was filtered through a pad of celite with a filter paper on top. The filtrate was concentrated and turned solid overnight. TLC and NMR revealed only minor impurities and the crude (1.256 g, 69%) was used without further purification. ^1^H NMR (400 MHz, Chloroform-*d*) δ 7.30–7.24 (m, 8H), 7.21–7.10 (m, 2H), 4.13 (q, *J* = 7.1 Hz, 4H), 2.59 (t, *J* = 7.5 Hz, 4H), 1.98–1.79 (m, 4H), 1.45 (tdd, *J* = 8.8, 6.0, 4.3 Hz, 4H), 1.19 (t, *J* = 7.1 Hz, 6H). ^13^C NMR (101 MHz, Chloroform-*d*) δ 171.8, 141.9, 128.5, 128.4, 126.0, 61.2, 57.5, 36.0, 31.8, 25.8, 14.2. HRMS-ESI: C_25_H_32_NaO_4_^+^ [M + Na]^+^ calcd: 419.2193, found: 419.2166.

*5,5-bis(3-phenylpropyl)pyrimidine-2,4,6(1H,3H,5H)-trione* (**4**). To a solution of urea (1.51 g, 25.22 mmol) in anhydrous DMF (10 ml) was slowly added NaH (151 mg, 6.3 mmol) and the resulting solution was stirred for 10 min before a solution of **3** (1.0 g, 2.522 mmol) in DMF (8 ml) was added. The resulting mixture was stirred overnight. The reaction was diluted with EtOAc (50 ml), washed with 10% citric acid sol. (3 × 30 ml), 10% NaHCO_3_ sol. (2 × 20 ml), and brine (30 ml). The organic phase was dried over Na_2_SO_4_, filtered and concentrated. The crude product was dissolved in CHCl_3_ and purified on automated flash chromatography affording **4** (595 mg, 65%) as a white powder. ^1^H NMR (400 MHz, CDCl_3_): δ 9.02 (s, 2H), 7.30–7.23 (m, 4H), 7.19 (t, *J* = 7.2 Hz, 2H), 7.11 (d, *J* = 7.4 Hz, 4H), 2.57 (t, *J* = 7.3 Hz, 4H), 2.25–1.87 (m, 4H), 1.73–1.15 (m, 4H). ^13^C NMR (101 MHz, DMSO-*d*_6_) δ 173.0, 149.8, 141.2, 128.3, 128.2, 125.9, 54.8, 37.7, 34.8, 26.4. HRMS-ESI: C_22_H_23_N_2_O_3_^−^ [M–H]^–^ calcd: 363.1714, found: 363.1706.

*1,3-bis(4-bromobutyl)-5,5-bis(3-phenylpropyl)pyrimidine-2,4,6(1H,3H,5H)-trione* (**5**). To a stirred solution of **3** (0.582 g, 1.6 mmol) in DMF (15 mL) was added Na_2_CO_3_ (1.32 g, 1.2 mmol, 6 equiv.) and 1,4-dibromobutane (1.88 mL, 1.6 mmol, 10 equiv.). The reaction mixture was stirred for 48 h, diluted with EtOAc (50 mL) and washed with 10% citric acid sol. (3 × 25 mL), 10% NaHCO_3_ sol. (2 × 25 mL), and brine (25 mL). The organic phase was dried over Na_2_SO_4,_ filtered and concentrated. The crude product was purified on automated flash chromatography affording **5** (0.705 g, 70%) as a white powder. ^1^H NMR (400 MHz, CDCl_3_): δ ^1^H NMR (400 MHz, Chloroform-*d*) δ 7.26 (dd, *J* = 8.0, 6.6 Hz, 4H), 7.21–7.14 (m, 2H), 7.12–7.05 (m, 4H), 3.90 (t, *J* = 7.2 Hz, 4H), 3.38 (t, *J* = 6.5 Hz, 4H), 2.54 (t, *J* = 7.7 Hz, 4H), 2.10–1.97 (m, 4H), 1.85 (dq, *J* = 8.8, 6.0 Hz, 4H), 1.79–1.66 (m, 4H), 1.48–1.30 (m, 4H).^13^C NMR (101 MHz, CDCl_3_): δ ^13^C NMR (101 MHz, Chloroform-*d*) δ 171.6, 150.6, 141.1, 128.6, 128.3, 126.2, 56.5, 41.2, 39.7, 35.7, 32.8, 30.0, 27.1, 26.8. HRMS-ESI: C_30_H_38_^79^Br^81^Br_2_N_2_O_3_ [M + Br^81^]^–^ calcd: 715.0397, found: 715.0388.

*1,3-bis(4-azidobutyl)-5,5-bis(3-phenylpropyl)pyrimidine-2,4,6(1H,3H,5H)-trione* (**6**). To a stirred solution of **5** (690 mg, 1.1 mmol) in 10 mL DMF was added NaN_3_ (210 g, 3.2 mmol, 3 equiv.) and stirred for 18 h. The reaction mixture was diluted with EtOAc (30 mL), washed with water (3 × 50 mL) and brine. The organic phase was dried over Na_2_SO_4_, filtered and concentrated to give the crude product **6** as white crystals (0.602 g, 99%). ^1^H NMR (400 MHz, CDCl_3_): δ 7.29–7.22 (m, 4H), 7.21–7.15 (m, 2H), 7.10–7.05 (m, 4H), 3.89 (t, *J* = 7.1 Hz, 4H), 3.27 (t, *J* = 6.6 Hz, 4H), 2.54 (t, *J* = 7.7 Hz, 4H), 2.07–1.97 (m, 4H), 1.71–1.51 (m, 8H), 1.43–1.32 (m, 4H). ^13^C NMR (101 MHz, CDCl_3_): δ 171.6, 150.5, 141.1, 128.5, 128.3, 126.2, 56.5, 50.9, 41.5, 39.7, 35.7, 27.0, 26.3, 25.3. HRMS-ESI: C_30_H_38_N_8_NaO_3_^+^ [M + Na]^+^ calcd: 581.2959, found: 581.2961.

4,4′-(2,4,6-trioxo-5,5-bis(3-phenylpropyl)dihydropyrimidine-1,3(2H,4H)-diyl)bis(butan-1-aminium) (MPM-1). To a stirred solution of 6 (574 mg, 1.03 mmol) and Et_3_N (0.30 mL, 2.1 equiv.) in i-PrOH:THF (1:1, 6 mL) was added 1,3-propanedithiol (0.212 mL, 2.05 equiv.). The mixture was stirred for 5 min before addition of NaBH_4_ (78 mg, 2 equiv.). After 48 h reaction time, Boc_2_O (90 mg, 0.41 mmol, 4 equiv.) was added and the reaction was stirred for 18 h and evaporated, before EtOAc (20 mL) and water (15 mL) were added and stirred for 1 h. The two phases were filtered using a glass funnel filter with a sinter glass disc. The organic phase was washed with water (3 × 15 mL) and brine (15 mL) and concentrated. The resulting crude was purified by automated flash chromatography and evaporated. The Boc-protected intermediate was deprotected with TFA (2 mL, 26 mmol) in CH_2_Cl_2_ (5 mL) for 18 h. The reaction mixture was concentrated, and the crude product purified by RP automated flash chromatography and lyophilized to give MPM-1 (268 mg, 53%) as the TFA-salt. ^1^H NMR (400 MHz, CD_3_OD): δ 7.27–7.21 (m, 4H), 7.18–7.13 (m, 2H), 7.11–7.07 (m, 4H), 3.95–3.87 (m, 4H), 2.97–2.90 (m, 4H), 2.54 (t, *J* = 7.4 Hz, 4H), 2.00–1.92 (m, 4H), 1.65 (p, *J* = 3.7 Hz, 7H), 1.40 (dq, *J* = 12.1, 7.6 Hz, 4H). ^13^C NMR (101 MHz, CDCl_3_): δ 172.9, 151.9, 142.5, 129.5, 129.3, 127.1, 57.6, 42.2, 40.3, 40.2, 36.4, 27.9, 26.0, 25.9. HRMS-ESI: C_30_H_43_N_4_O_3_^+^ [M + H]^+^ calcd: 507.3330, found: 507.3329.

### Cell lines and cell culture

The glioblastoma GL261-Luc2 cell line was kindly gifted by Dr. Adrienne Scheck. Wild type HeLa and ATG7 KO HeLa cells were a kind gift from Professor Terje Johansen. A375 (RRID:CVCL_0132) was obtained from Public Health England (PHE Culture Collection, London, UK). HSC-3 (RRID: CVCL_1288) was obtained from the Japanese Collection of Research Bioresources Cell Bank (JCRB Cell Bank, Osaka, Japan). PBMCs were isolated from blood samples from randomized anonymous healthy volunteers. The remaining cell lines, B16F1 (RRID:CVCL_0158), HepG2 (RRID:CVCL_0027), Jurkat (RRID:CVCL_0367), Ramos (RRID:CVCL_0597), HT-29 (RRID: CVCL_0320), MCF-7 (RRID: CVCL_0031), SK-N-AS (RRID:CVCL_1700), HUVEC (RRID:CVCL_2959) and MRC-5 (RRID:CVCL_0440) were all obtained from the American Type Culture Collection (ATCC, Manassas, VA, USA). Cells were kept at 37ºC with 5% CO_2_ and cultured in complete medium unless otherwise stated. For A375, B16F1, GL261-Luc2, HepG2, HeLa (wild type and ATG7 KO) and HSC-3 this consisted of high glucose Dulbecco’s Modified Eagle’s Medium (DMEM, Sigma-Aldrich) supplemented with 10% fetal bovine serum (FBS) and 1% L-glutamine (Sigma-Aldrich). Jurkat, Ramos, PBMCs, HT-29, MCF-7 and SK-N-AS were kept in RPMI-1640 (Sigma-Aldrich) supplemented with 10% FBS. MRC-5 was kept in Minimum Essential Medium Eagle (MEM, Sigma-Aldrich) with 10% FBS and HUVEC was kept in complete EGM™-2 Endothelial Cell Growth Medium-2 BulletKit™ (Lonza, Basel, Switzerland).

### MTS cytotoxicity assay

A colorimetric proliferation assay, based on the conversion of a tetrazolium compound (3-(4,5-dimethylthiazol-2-yl)-5-(3-carboxymethoxyphenyl)-2-(4-sulfophenyl)-2H-tetrazolium, inner salt; MTS) to a formazan product, was used to assess the cytotoxic effect of MPM-1. Cells were seeded at approximately 80% confluence in flat-bottom 96-well plates. For adherent cell lines, this corresponded to 2 × 10^4^ cells/well, which were left to adhere overnight. HeLa cells (wild type and ATG7 KO) were seeded at 1.5 × 10^4^ cells/well. Before treatment with MPM-1, cells were washed twice with serum free medium. Suspension cells were seeded on the same day as the experiment, in serum free medium. For Ramos and Jurkat, 8 × 10^4^ cells were seeded/well, and for PBMCs, 15 × 10^4^ were seeded/well. For determination of IC50 values, all cells were treated with MPM-1 in 100 μL serum free medium in a two-fold serial dilution series with concentrations ranging from 128 μg/μL to 0.125 μg/μL. For the viability assays with Bafilomycin A1, HSC-3 cells were pre-treated with 50 μL Bafilomycin A1 (100 nM) (Merck, Darmstadt, Germany) for one hour. Next, 50 μL of MPM-1 diluted in Bafilomycin A1 containing media was added to yield a final volume of 100 μL and a final concentration of MPM-1 of 8.5 or 17 μM. Serum free medium ± 1% Triton X-100 functioned as positive and negative controls, respectively. After four hours of incubation, 20 μL of MTS solution (CellTiter 96^®^ Aqueous One Solution, Promega, Madison, WI, USA) was added to each well and the plate was incubated for another 75 min. Absorbance was measured at 490 nm with a VersaMax™ Microplate reader (Molecular Devices, San Jose, CA, USA). The percentage of live cells was determined according to the formula:

$$\%=\frac{\mathrm{Abs\,} \mathrm{treated\,} \mathrm{sample}-\mathrm{Abs\,} \mathrm{positive\,} \mathrm{control}}{\mathrm{Abs\,} \mathrm{negative\,} \mathrm{control}-\mathrm{Abs\,} \mathrm{positive\,} \mathrm{control}} \times 100.$$

Each experiment was run three times with triplicate wells and the mean IC50 value was calculated for each cell line.

### Hemolysis assay

The hemolytic effect of MPM-1 was determined by the use of a hemolysis assay as previously described^39^. Briefly, human red blood cells were isolated and resuspended in PBS. Next, they were mixed with MPM-1 in PBS at varying concentrations. The concentration of red blood cells was 1% and the concentrations of MPM-1 ranged up to 500 µM. 0.1% Triton X-100 and pure PBS were used as positive and negative controls, respectively. After 1 h of incubation at 37 °C with agitation, the samples were centrifuged at 4000 rpm for 5 min and the supernatant was collected. The absorption of the supernatant was measured at 405 nm and the percentage of hemolysis was calculated using the same formula as for the MTS assay.

### Live cell imaging

HSC-3 cells were seeded, 1 × 10^5^ or 1.5 × 10^5^ cells per well, on glass-bottom 24-well plates that had been pre-coated with fibronectin and left to adhere overnight. This corresponded to 50,000 and 75,000 cells/cm^2^, respectively. Cells were washed in complete DMEM and stimulated with MPM-1 diluted in complete DMEM to 4.3, 8.5 or 17.0 μM. Upon addition of MPM-1 to the cells, the culture plate was incubated in a Celldiscoverer 7 (Zeiss, Oberkochen, Germany), which was set to take pictures of each well approximately every three minutes for a total of 23 h.

### Transmission electron microscopy

HSC-3 cells were seeded, 3 × 10^5^ cells per dish, in 35 mm dishes with a 14 mm gridded coverslip (MatTek, Ashland, MA, USA) that had been pre-coated with fibronectin and left to adhere overnight. Cells were washed in complete DMEM and stimulated with MPM-1 diluted in complete DMEM to 8.5 μM for 2 h or 6 h. One well was left untreated in complete DMEM alone. All processing was done in a microwave processor with a temperature control unit (Ted Pella, Redding, CA, USA). The cells were fixed for 14 min in a fixative containing 4% formaldehyde, 0.5% glutaraldehyde, and 0.05% malachite green in PHEM buffer (60 mM PIPES, 25 mM HEPES, 10 mM EGTA, 4 mM MgSO_4_·7H2O) (2 min vacuum on–off-on–off-on–off–on, 100 W) and subsequently washed twice with PHEM buffer. Post-fixation was done with 1% Osmium tetroxide and 1% K_3_Fe(CN)_6_ in 0.1 M cacodylic acid buffer. The cells were post-stained with 1% tannic acid and 1% uranyl acetate. Samples were then dehydrated in an increasing ethanol series (30–60–96–100%) and embedded in an epon equivalent (Agar). 70 nm sections were cut using a diamond knife (DiATOME, USA) on a UC7 ultramicrotome (Leica Microsystems, Wetzlar, Germany) and picked up on formvar-coated cupper grids. Sections were imaged using a Hitatchi HT7800 Transmission Electron Microscopy (Hitachi, Tokyo, Japan) with a XAROSA camera (EMSIS GmbH, Münster, Germany).

### Scanning electron microscopy

HSC-3 cells were seeded at 1.5 × 10^5^ cells per well, on fibronectin coated glass coverslips that were placed at the bottom of a 24-well plate. Cells were washed in complete DMEM and stimulated with MPM-1 diluted in complete DMEM to 8.5 μM for 2 or 6 h. One well was left untreated in complete DMEM alone. Processing was performed as described for the transmission electron microscopy samples up until the last step of the dehydration series (100% ethanol). At this point, samples were dehydrated by incubation 3 × 2 min in hexamethyldisilazane (Sigma-Aldrich). The samples were mounted on specimen holders and coated with gold–palladium in a Polaron Sputter Coater (Quorum Technologies, Lewes, UK) before being imaged on a GeminiSEM 360 (Zeiss).

### Confocal microscopy

HSC-3 cells were seeded at 5 × 10^4^ cells/well, in an 8-well chambered coverglass that had been pre-coated with fibronectin. The following day, cells were washed once in complete medium and then treated with 8.5 µM MPM-1 in 350 µL for 1 h, 2 h, 4 h or 6 h. One well was left untreated.

For staining of p62 and LC3B, cells were fixed in 4% formaldehyde in PHEM buffer and left at 4 °C until the next day. Cells were permeabilized by incubating them in 5% methanol in PBS for 5 min on ice. Next, cells were washed twice in PBS and blocked by 45 min incubation in PBS 3% goat serum before they were incubated for 60 min with primary antibodies targeting p62 (#GP62‐C, guinea pig polyclonal, Progen, diluted 1:2000) and LC3B (#L7543, rabbit polyclonal, Sigma‐Aldrich, diluted 1:1000) in PBS 1% goat serum. The cells were then washed 6 × 2 min in PBS before being incubated with secondary antibodies (Alexa Fluor Plus 555 conjugated goat anti-rabbit (#A32732, Thermo Fisher), and Alexa Fluor 488 conjugated goat anti-guinea pig (#A11073, Thermo Fisher) diluted 1:1000 for 30 min. The cells were then washed 4 × 2 min in PBS before being incubated with DAPI (Thermo Fisher) (1 µg/mL in PBS) for 5 min followed by 2 × 2 min washing in PBS.

For staining of lysosomes, lysotracker Deep Red (L12492, Thermo Fisher) was included in each well for the last 30 min of incubation at a final concentration of 50 nM. Cells were then fixed in 4% formaldehyde for 15 min at room temperature. Next, cells were washed 4 × 2 min in PBS before being incubated with DAPI (1 µg/mL in PBS) for 5 min followed by 2 × 2 min washing in PBS.

Imaging was performed on a LSM 780 confocal microscope (Zeiss) and analysis was performed in Volocity ver 6.3 (PerkinElmer).

### Flow cytometric apoptosis detection

The mode of death induced by MPM-1 was investigated with an apoptosis detection kit (88-8005-74, Thermo Fisher Scientific, Waltham, MA, USA), which combines staining with FITC-labeled Annexin V and propidium iodide (PI). HSC-3 cells were seeded, 4 × 10^5^ cells/well in 6-well plates, and left to adhere overnight. The following day, one well was treated with 100 nM Staurosporine. On day two, the remaining wells were treated with 8.5 or 17.0 μM MPM-1 for up to four hours. To retain cells that could have detached from the well, the supernatant from each well was transferred to microcentrifuge tubes. The remaining cells were trypsinized and mixed with their respective supernatants. Ramos cells were seeded on the day of analysis, 6 × 10^5^ cells/well in 24-well plates. Cells were treated with 2 μM TBTC for 2 h, or 7.5 or 15 μM MPM-1 for up to four hours. HSC-3 and Ramos cells were centrifuged and washed in binding buffer before being stained with the Annexin V-FITC antibody at 1:20 dilution for 15 min. Next, cells were washed in binding buffer again and transferred to flow cytometry tubes, before being stained with PI at 1:150 dilution for at least five minutes before analysis.

### Flow cytometric analysis of mitochondrial membrane potential

Changes in the mitochondrial membrane potential were analyzed with the fluorescent mitochondrial dye TMRE (T669, Thermo Fisher Scientific). HSC-3 cells were seeded, 6 × 10^5^ cells/well in 6-well plates, and left to adhere overnight. Cells were washed in serum free RPMI and treated with 1 μM staurosporine for four hours, or 8.5 or 17.0 μM MPM-1 for up to four hours. Ramos cells were seeded on the day of the experiment, 6 × 10^5^ cells/well in serum free RPMI in 24-well plates, and treated with 2 μM TBTC for two hours, or 7.5 or 15 μM MPM-1 for up to four hours. 20 min before incubation was ended, TMRE was added to a final concentration of 5 nM for both cell lines. HSC-3 cells were washed in PBS, trypsinized and resuspended in PBS 2% FBS before analysis. Ramos cells were washed in PBS 2% FBS and analyzed directly.

### Flow cytometric detection of calreticulin exposure

For detection of cell surface exposure of calreticulin, HSC-3 cells were seeded at 1.5 × 10^5^ cells/well in a 24-well plate and left to adhere overnight. Cells were washed in complete DMEM and stimulated with MPM-1 diluted in complete DMEM to 8.5 or 17 μM for 4 h. Next, the cells were washed in PBS, trypsinized and resuspended in PBS 2% FBS before being stained with an Alexa Fluor 647 conjugated anti-calreticulin antibody (#ab196159, Abcam, Cambridge, United Kingdom) at 1:50 dilution. After 40 min incubation, cells were washed and resuspended in PBS 2% FBS, stained with PI at 1:150 dilution for at least five minutes, and immediately analyzed by flow cytometry.

All flow cytometric analyses in the present study were performed on a BD LSRFortessa™ (Becton Dickinson, Franklin Lakes, NJ, USA). Analyses were performed in FlowJo™ v.10 (https://www.flowjo.com/).

### Luminescence based detection of ATP release

Release of ATP from cells treated with MPM-1 was detected with an ATP determination kit (A22066, Thermo Fisher Scientific) according to the manufacturer’s protocol. HSC-3 cells were seeded at 2 × 10^4^ cells/well in flat-bottom 96-well plates and left to adhere overnight. Before treatment with MPM-1, cells were washed twice with serum free RPMI. Ramos cells were seeded on the same day as the experiment at 8 × 10^4^ cells/well in serum free RPMI in flat-bottom 96-well plates. HSC-3 and Ramos cells were stimulated with 8.5 or 17.0 μM (HSC-3) or 7.5 or 15 μM (Ramos) MPM-1 in a total volume of 100 μL for 30 min, 1 h or 2 h. After stimulation, 70 μL of the supernatant was carefully removed from each well and mixed well before 10 μL was transferred to wells on a white flat-bottom 96-well plate. The plate was inserted into the CLARIOstar microplate reader (BMG LABTECH, Ortenberg, Germany), which was set to add 90 μL of pre-made reaction buffer to each well and subsequently record luminescence. Luminescence was measured at 555–570 nm for 10 s. ATP release was expressed as fold increase of the luminescence in untreated samples.

### Detection of HMGB1 release by western blotting

Release of HMGB1 from cells treated with MPM-1 was detected by Western blotting. Ramos cells were suspended in serum free RPMI and seeded at 6 × 10^5^ cells/well, in a 24-well plate before being treated with 7.5 μM MPM-1 in a total volume of 750 μL. HSC-3 cells were seeded at 4 × 10^5^ cells/well, in a 6-well plate, and left to adhere overnight. Cells were then washed once with serum free RPMI and treated with 17 μM MPM-1 in a total volume of 1 mL. Ramos and HSC-3 cells were treated for 0.5, 1, 2, 3 or 4 h in separate wells. Serum free medium ± 1% Triton X-100 functioned as positive and negative controls, respectively. After treatment, supernatants were collected and centrifuged to remove cell debris before being mixed with DTT and sample buffer. The samples were boiled for 5 min and loaded on a NuPAGE^®^ 10% Bis–Tris Gel (Thermo Fisher Scientific) before being electro-transferred to a polyvindiline dilfluoride (PVDF) immobilon-P membrane (Merck, Darmstadt, Germany). The membrane was blocked for 1 h with 5% non-fat dry milk in TBST and then incubated overnight at 4 °C with the primary antibody targeting HMGB1 (Abcam, #ab18256) diluted 1:1000 in 5% non-fat dry milk in TBST. Next, the membrane was washed and incubated with a horseradish peroxidase-conjugated goat anti-rabbit secondary antibody (Southern Biotech, Birmingham, AL, USA, Cat #4050-05) diluted 1:2000 in 5% non-fat dry milk in TBST for 1 h. After washing, the membrane was incubated for 5 min with 5 mL pre-mixed chemiluminescent peroxidase substrate-3 (Merck) and subsequently imaged on an ImageQuant LAS 3000 (GE Healthcare, Chicago, IL, USA). Band intensities were analyzed in Image Studio Lite Ver 5.2 (https://www.licor.com/bio/image-studio-lite/). HMGB1 release was expressed as percentage of release relative to the positive control sample.

### Statistical analyses

Statistical analyses were performed in GraphPad Prism 9.0 (https://www.graphpad.com/). A p-value of < 0.05 was considered statistically significant. In all graphs, asterisks indicate significant differences: *p < 0.05, **p < 0.01, ***p < 0.001, ****p < 0.0001.

### Ethical considerations

All use of human material was according to national guidelines. Blood samples from randomized anonymous healthy volunteers were obtained from the blood bank at the University Hospital North Norway in Tromsø, which is officially approved by the Norwegian Directorate of Health. Donors had given written informed consent for use of their blood for research, in accordance with the Declaration of Helsinki. Additional ethical approval for the use of anonymous blood samples for research was not required according to the Norwegian Health Research Act.

## Supplementary Information

Supplementary Information 1.

Supplementary Information 2.

Supplementary Video 1.

Supplementary Video 2.

## Data Availability

The datasets generated and analyzed during the current study are available from the corresponding author on reasonable request.
