# Supplementary material for: The marine natural product mimic MPM-1 is cytolytic and induces DAMP release from human cancer cell lines
Source: Sci Rep. 2022 Sep 16;12:15586. doi: 10.1038/s41598-022-19597-4 (PMC9481558; doi:10.1038/s41598-022-19597-4)
Supplement: Supplementary file 1 — Supplementary Information 1. [file 41598_2022_19597_MOESM1_ESM.pdf]

**Supplementary Data S1**

NMR for all intermediate molecules produced during the synthesis of the target compound MPM-1.

Compound 2

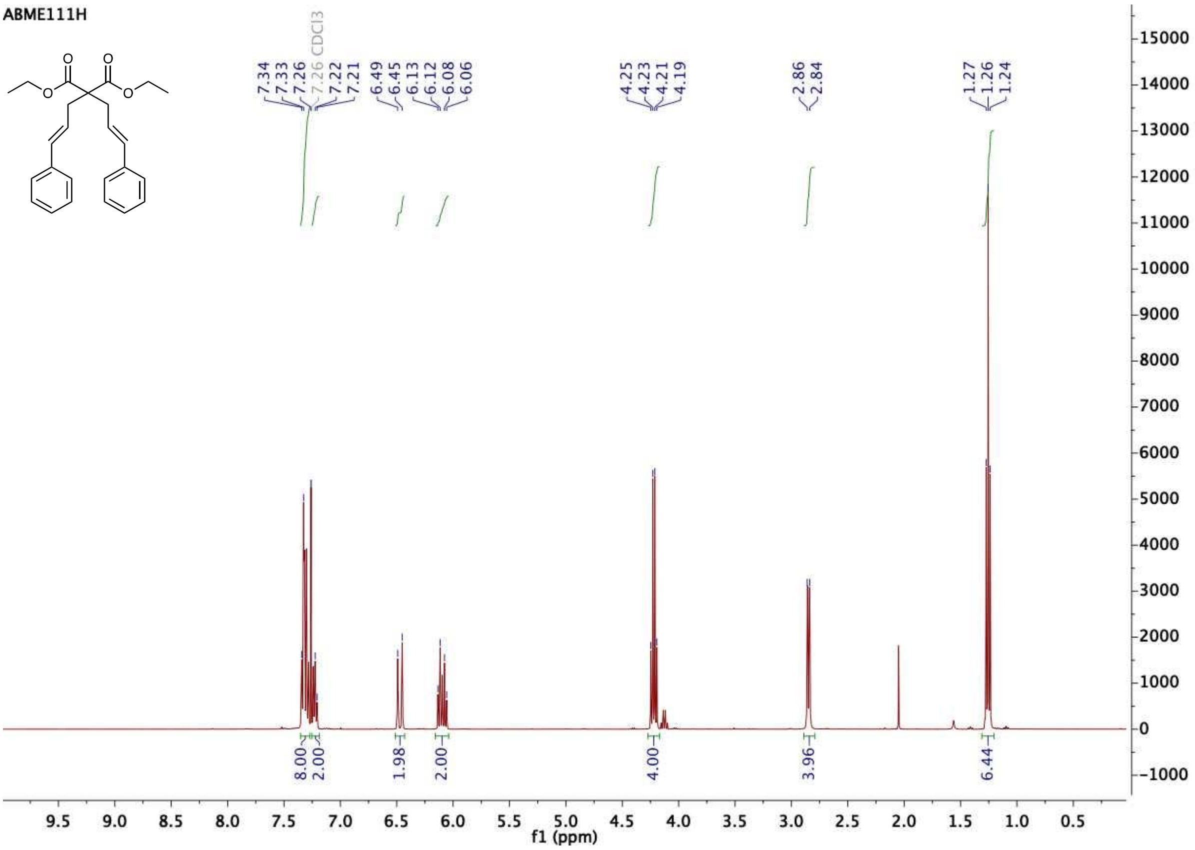

Compound 3

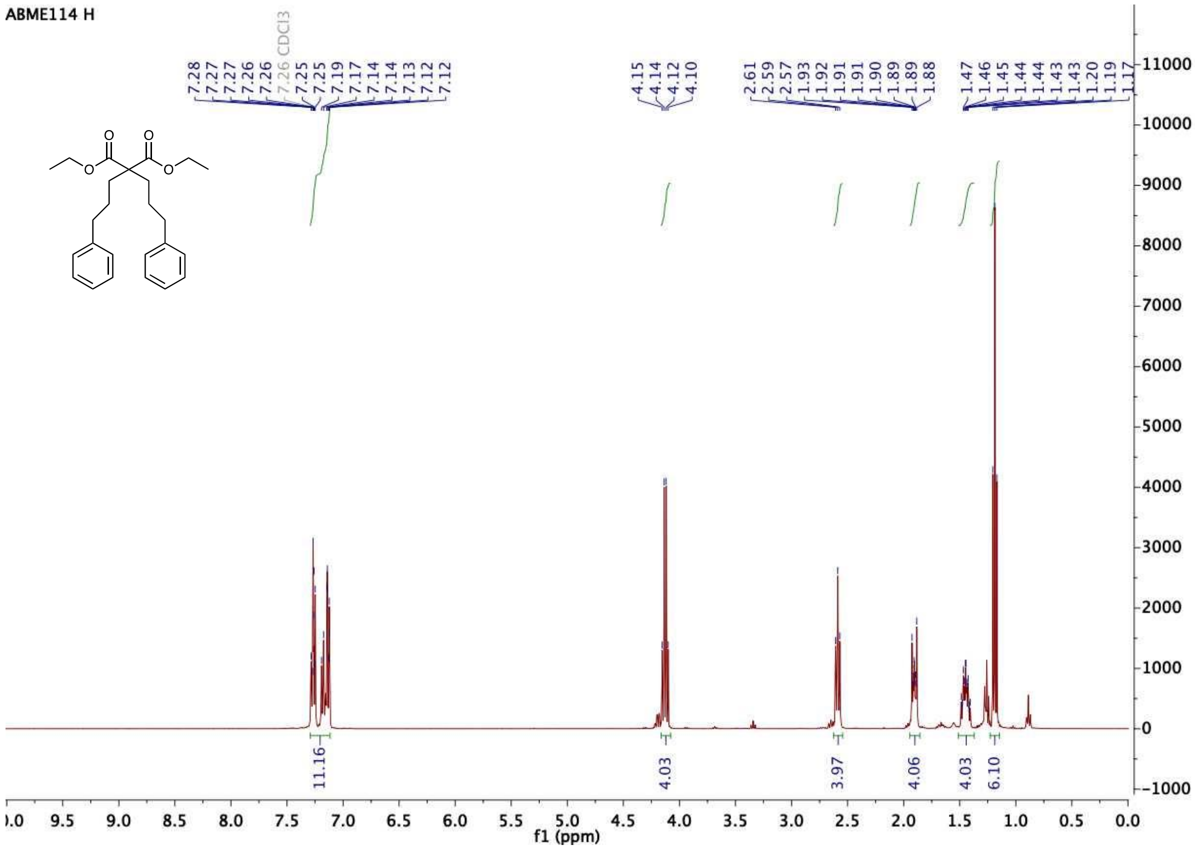
ABME114 H
7.28 7.27 7.27 7.26 7.26 7.26 CDCl3 7.25 7.25 7.19 7.17 7.14 7.14 7.13 7.12 7.12
4.15 4.14 4.12 4.10
2.61 2.59 2.57 1.93 1.92 1.91 1.91 1.90 1.89 1.89 1.88
1.47 1.46 1.45 1.44 1.44 1.43 1.43 1.20 1.19 1.17
11.16
4.03
3.97
4.06
4.03
6.10
f1 (ppm)

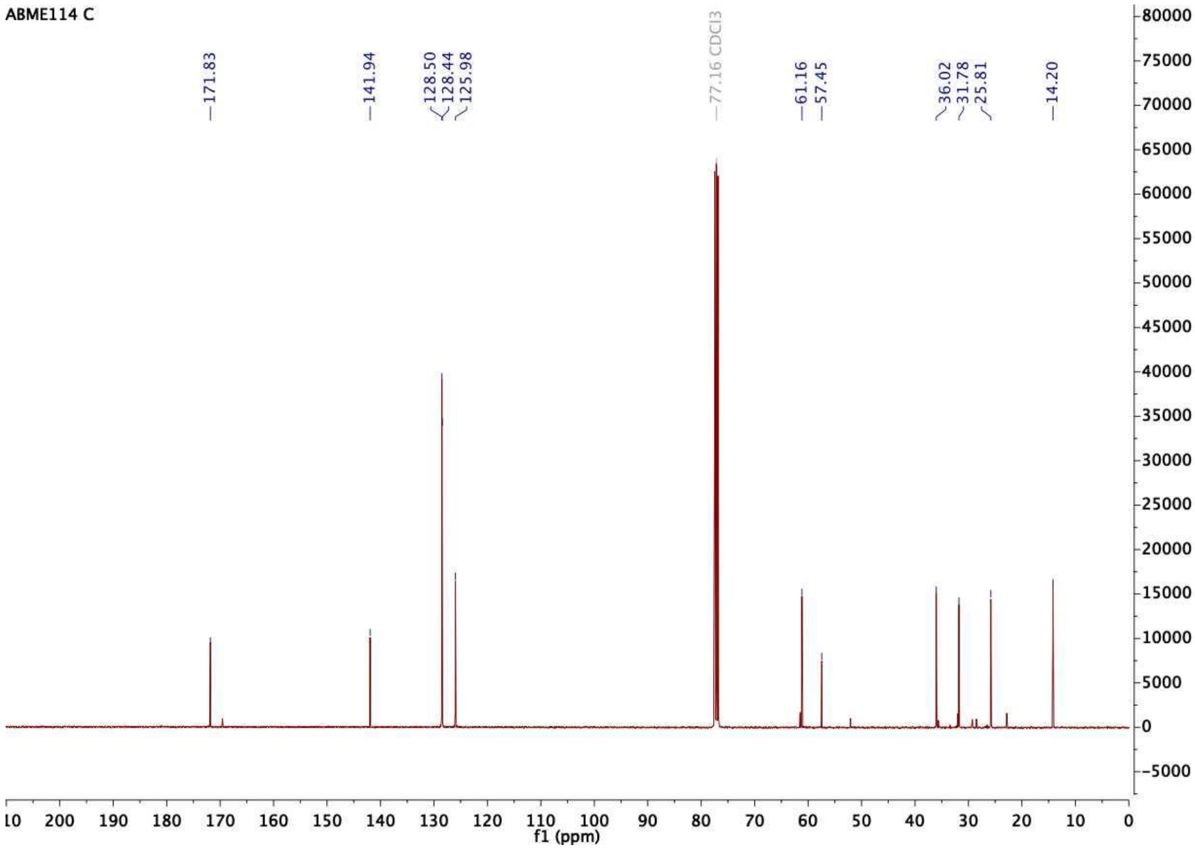
ABME114 C
171.83
141.94
128.50
128.44
125.98
77.16 CDCl3
61.16
57.45
36.02
31.78
25.81
14.20
f1 (ppm)

Compound 4

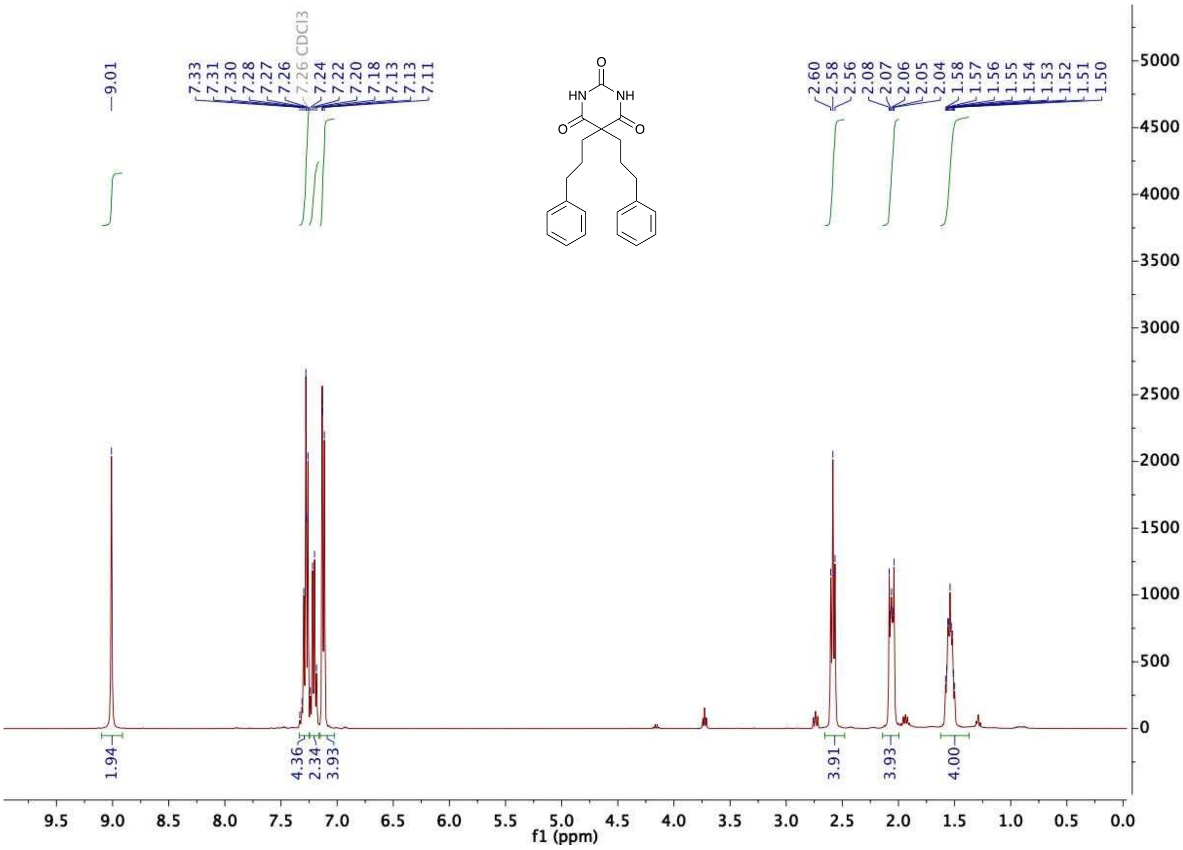

9.01
7.33 7.31 7.30 7.28 7.27 7.26 7.26 CDCl3 7.24 7.22 7.20 7.18 7.13 7.13 7.11
2.60 2.58 2.56 2.08 2.07 2.06 2.05 2.04 1.58 1.57 1.56 1.55 1.54 1.53 1.52 1.51 1.50
O
HN
NH
O
O
1.94
4.36 2.34 3.93
3.91
3.93
4.00
f1 (ppm)


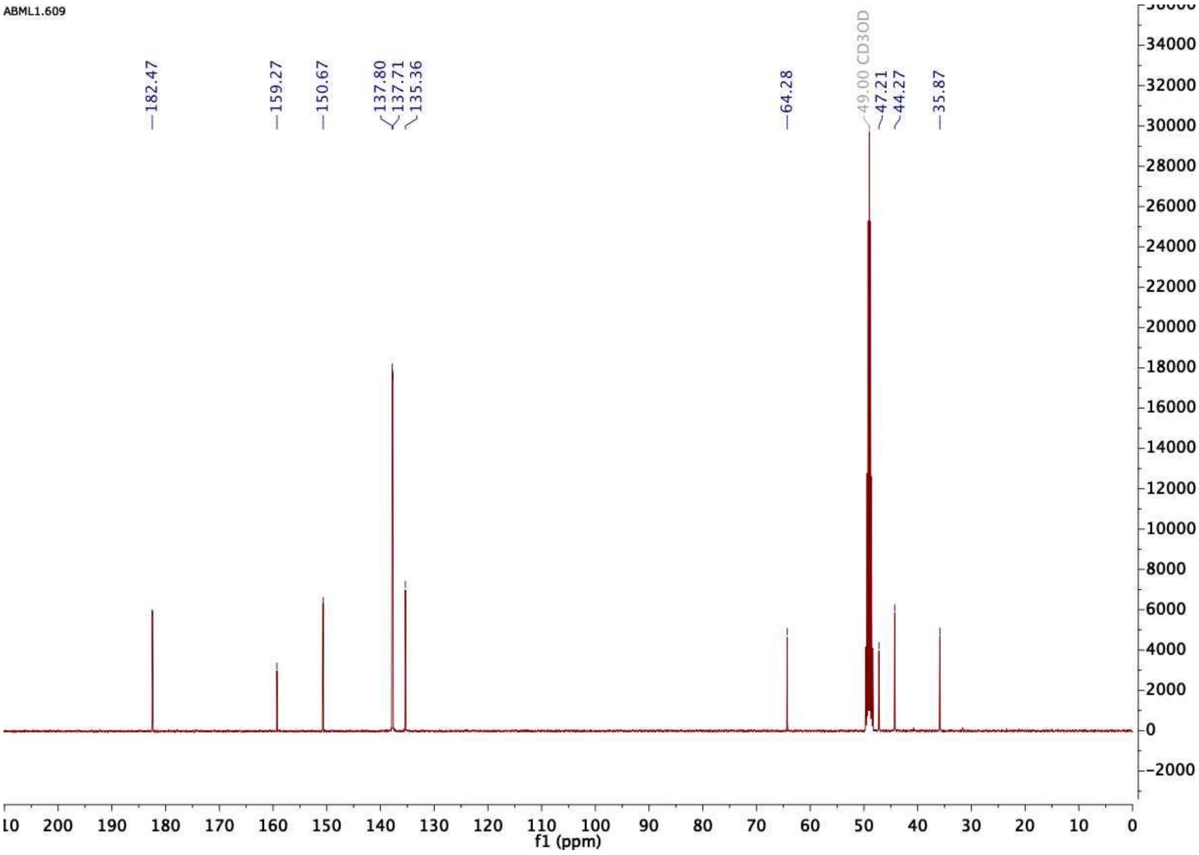

ABML1.609
182.47
159.27
150.67
137.80 137.71 135.36
64.28
49.00 CD3OD
47.21
44.27
35.87
f1 (ppm)

Compound 5

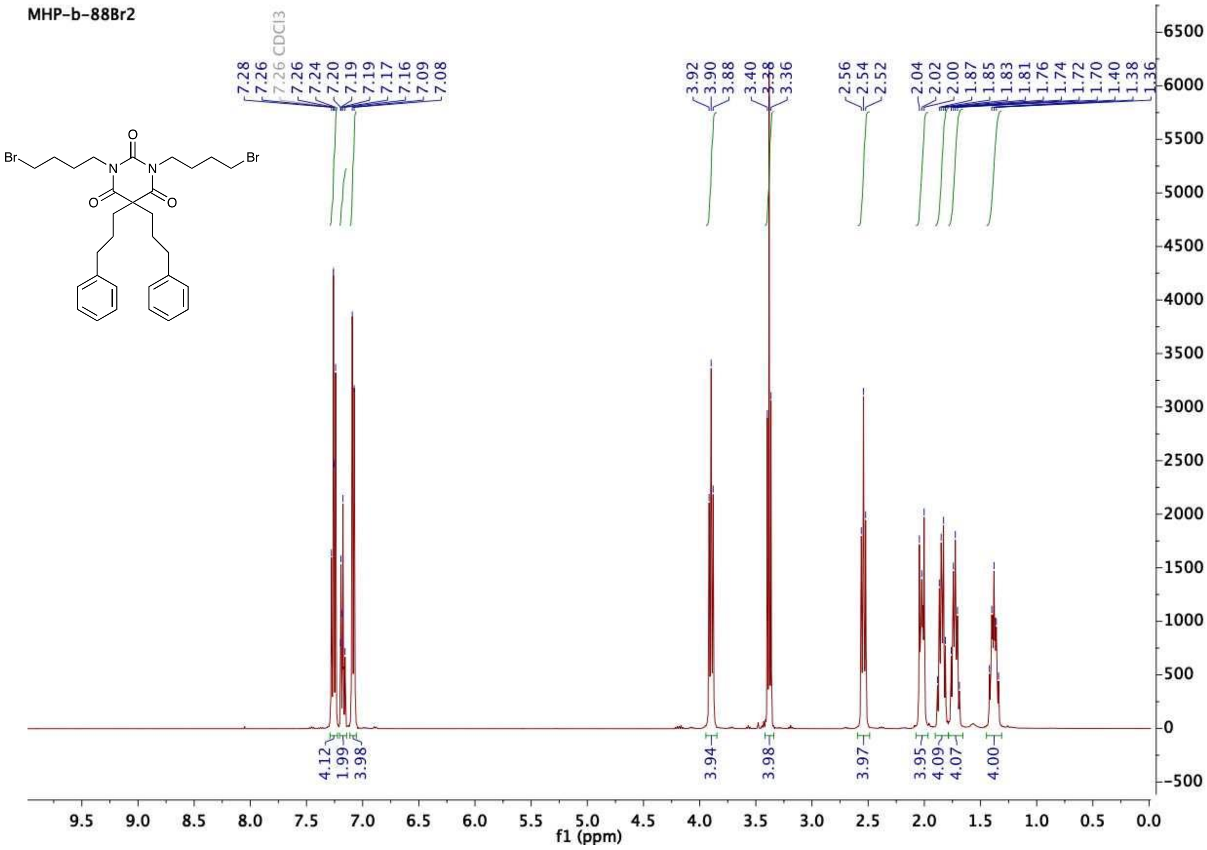

MHP-b-88Br2
Br
N
N
Br
O
O
O
7.28
7.26
7.26 CDCl3
7.26
7.24
7.20
7.19
7.19
7.17
7.16
7.09
7.08
3.92
3.90
3.88
3.40
3.38
3.36
2.56
2.54
2.52
2.04
2.02
2.00
1.87
1.85
1.83
1.81
1.76
1.74
1.72
1.70
1.40
1.38
1.36
4.12
1.99
3.98
3.94
3.98
3.97
3.95
4.09
4.07
4.00
f1 (ppm)


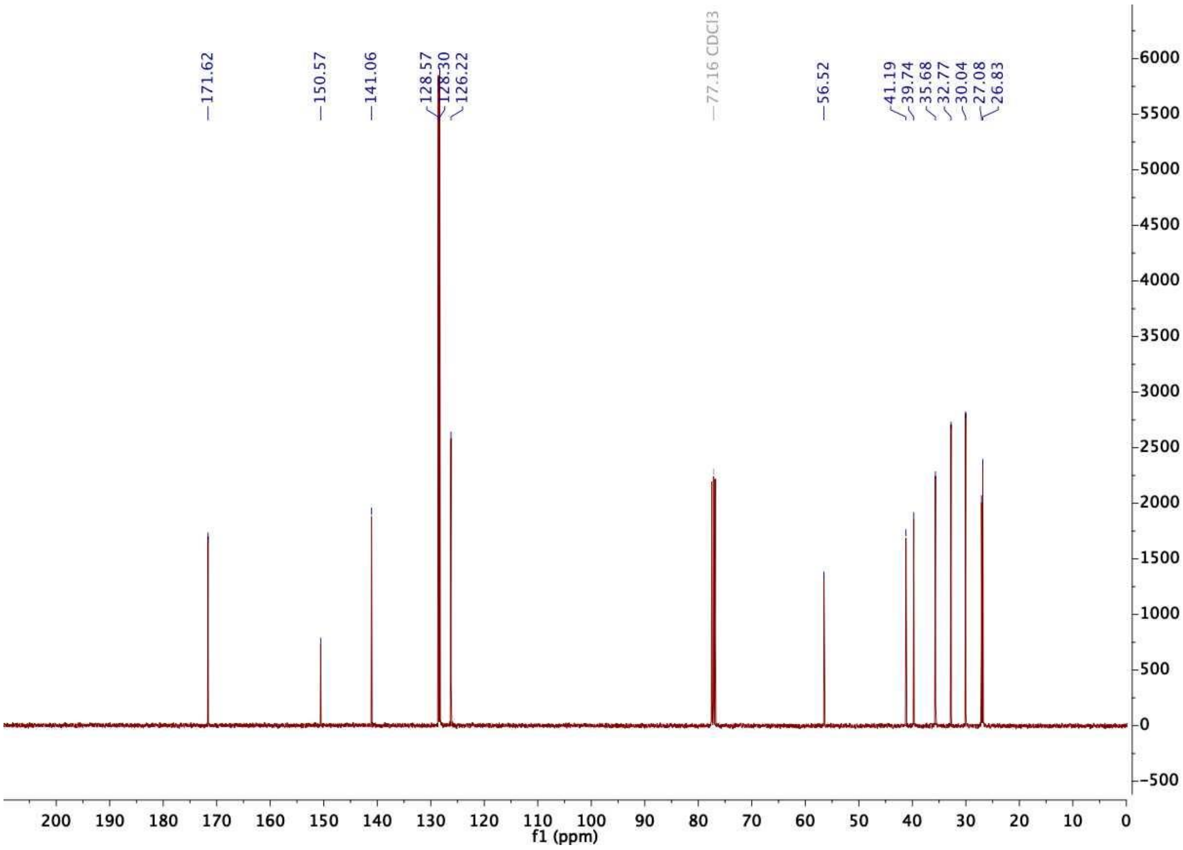

171.62
150.57
141.06
128.57
128.30
126.22
77.16 CDCl3
56.52
41.19
39.74
35.68
32.77
30.04
27.08
26.83
f1 (ppm)

Compound 6

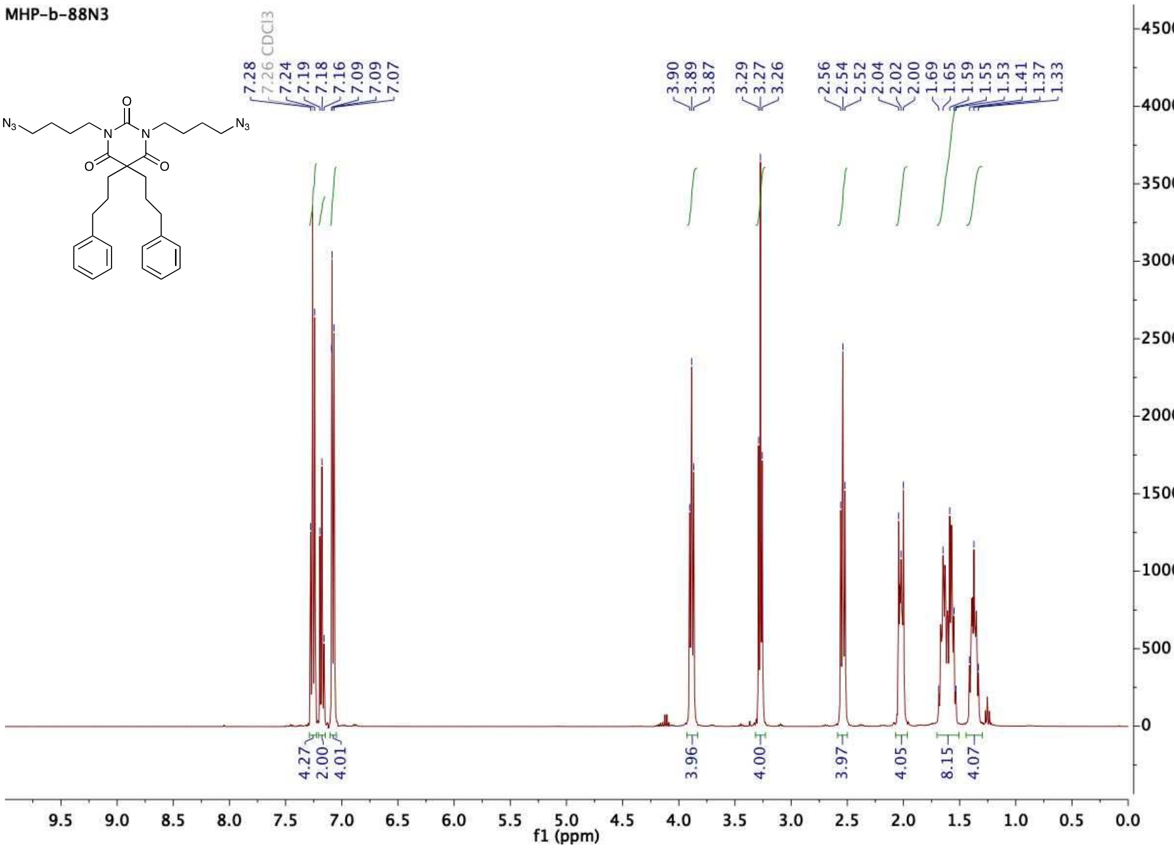
MHP-b-88N3
7.28
7.26 CDCl3
7.24
7.19
7.18
7.16
7.09
7.09
7.07
3.90
3.89
3.87
3.29
3.27
3.26
2.56
2.54
2.52
2.04
2.02
2.00
1.69
1.65
1.59
1.55
1.53
1.41
1.37
1.33
4.27
2.00
4.01
3.96
4.00
3.97
4.05
8.15
4.07
f1 (ppm)

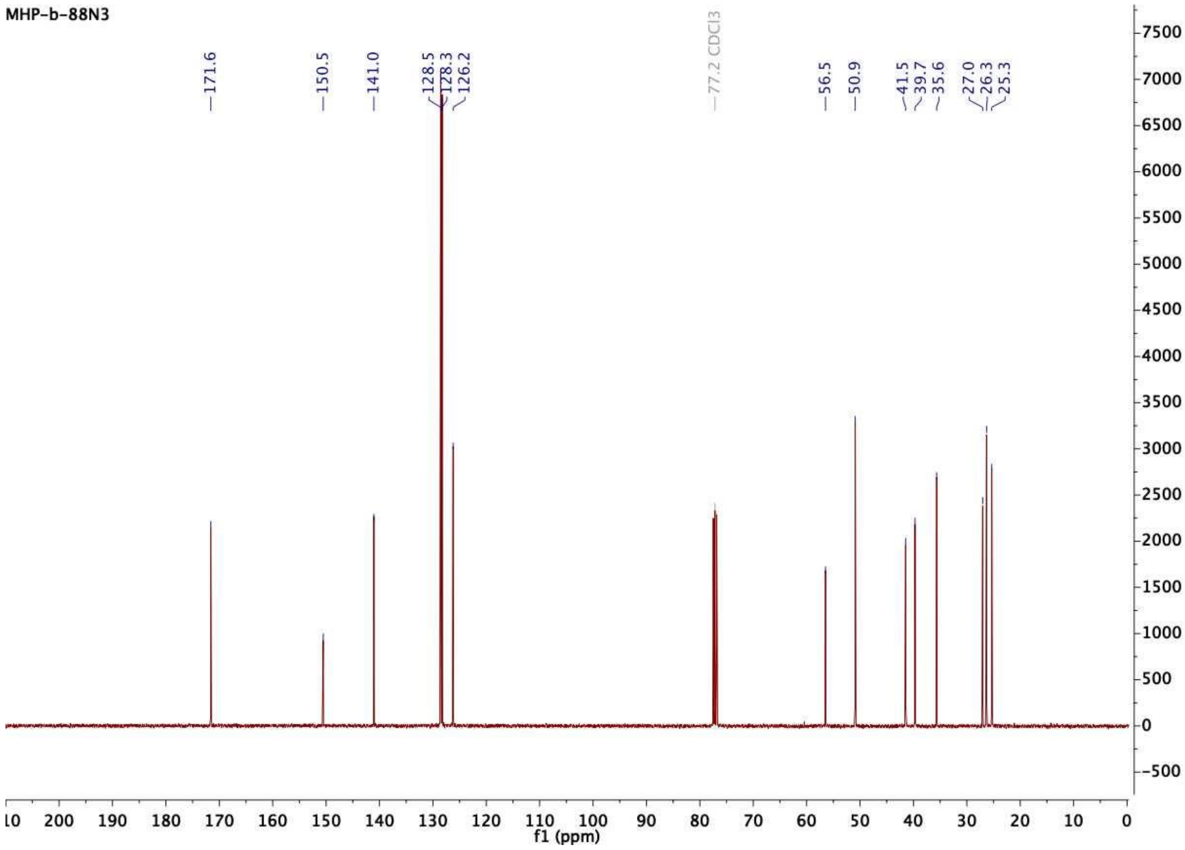
MHP-b-88N3
171.6
150.5
141.0
128.5
128.3
126.2
77.2 CDCl3
56.5
50.9
41.5
39.7
35.6
27.0
26.3
25.3
f1 (ppm)

MPM-1

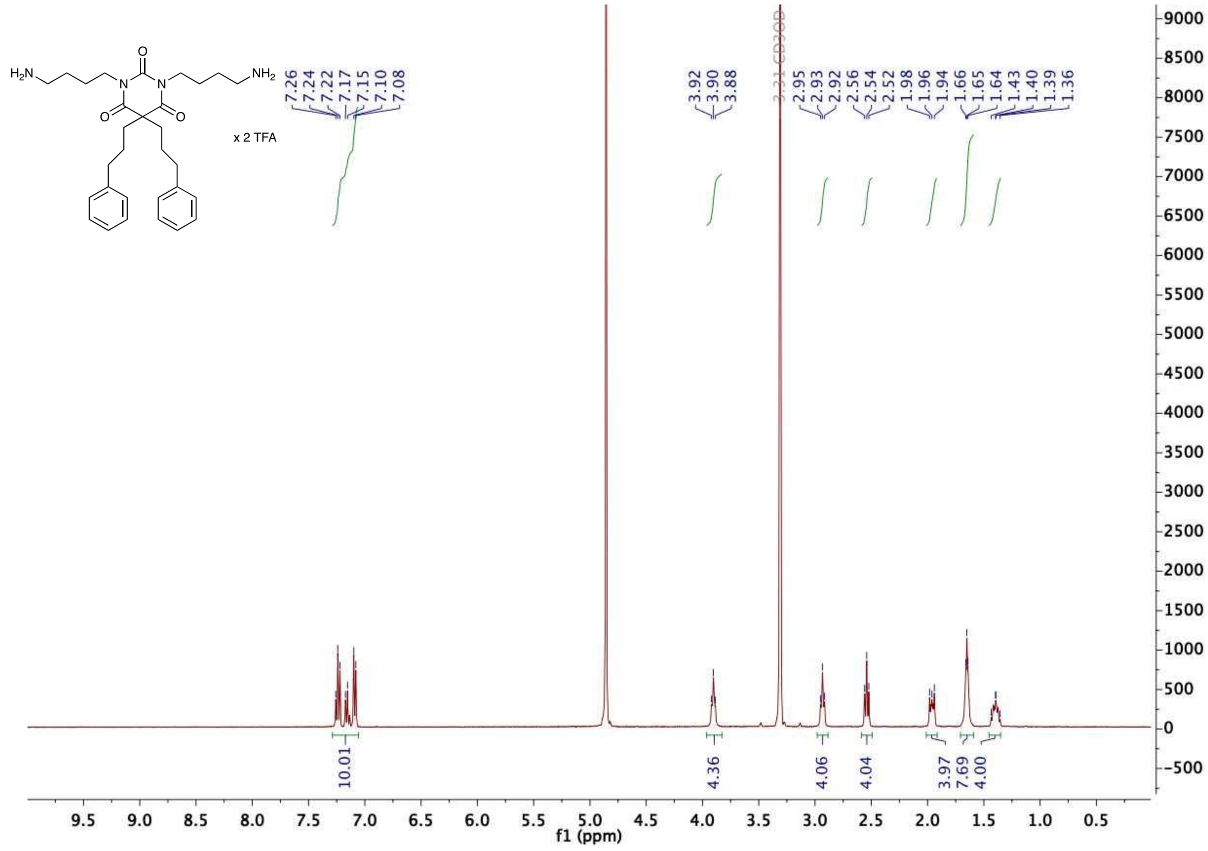

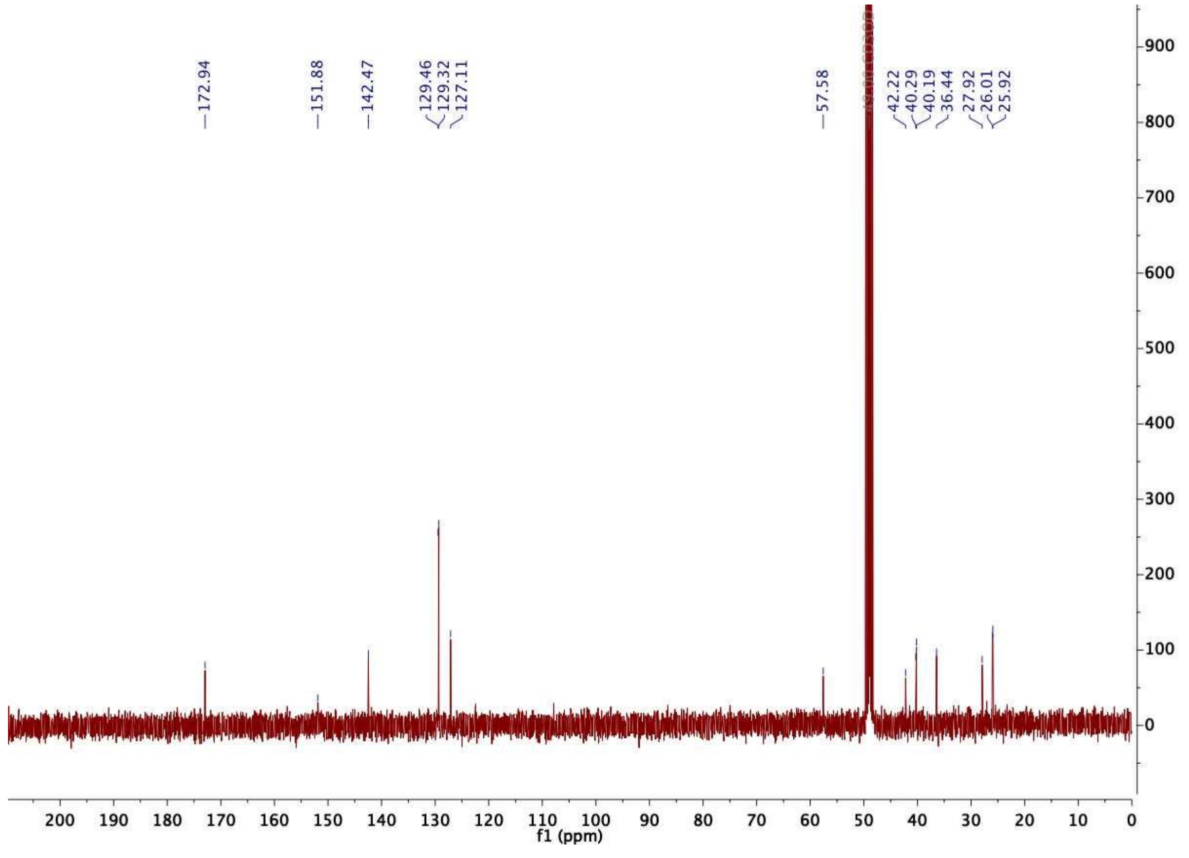

Purity SFC$^2$

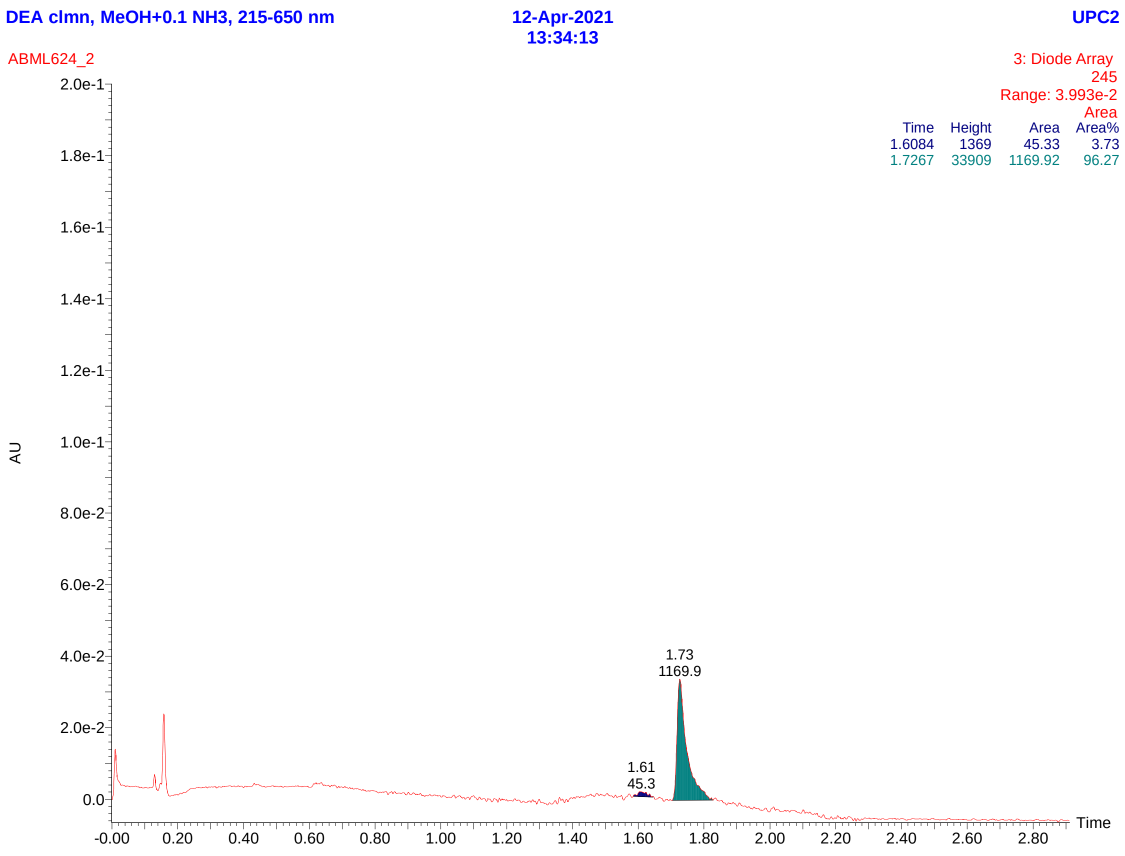
DEA clmn, MeOH+0.1 NH3, 215-650 nm
12-Apr-2021
13:34:13
UPC2
ABML624_2
3: Diode Array
245
Range: 3.993e-2
Area
Time Height Area Area%
1.6084 1369 45.33 3.73
1.7267 33909 1169.92 96.27
1.73
1169.9
1.61
45.3
AU
Time
