# Supplementary material for: The marine natural product mimic MPM-1 is cytolytic and induces DAMP release from human cancer cell lines
Source: Sci Rep. 2022 Sep 16;12:15586. doi: 10.1038/s41598-022-19597-4 (PMC9481558; doi:10.1038/s41598-022-19597-4)
Supplement: Supplementary file 2 — Supplementary Information 2. [file 41598_2022_19597_MOESM2_ESM.pdf]

The Marine Natural Product Mimic MPM-1 is Cytolytic and Induces DAMP Release from Human Cancer Cell Lines

Susannah von Hofsten[1*], Marianne Hagensen Paulsen[2], Synnøve Norvoll Magnussen[1], Dominik Ausbacher[2], Mathias Kranz[3], Annette Bayer[4], Morten B. Strøm[2] and Gerd Berge[1]

[1]Department of Medical Biology, Faculty of Health Sciences, UiT The Arctic University of Norway, 9037 Tromsø, Norway. [2]Department of Pharmacy, Faculty of Health Sciences, UiT The Arctic University of Norway, 9037 Tromsø, Norway. [3]PET Imaging Center Tromsø, University Hospital of North Norway, 9019 Tromsø, Norway. [4]Department of Chemistry, UiT The Arctic University of Norway, 9037 Tromsø, Norway. *email: susannah.hofsten@uit.no

Supplementary Figures and Legends

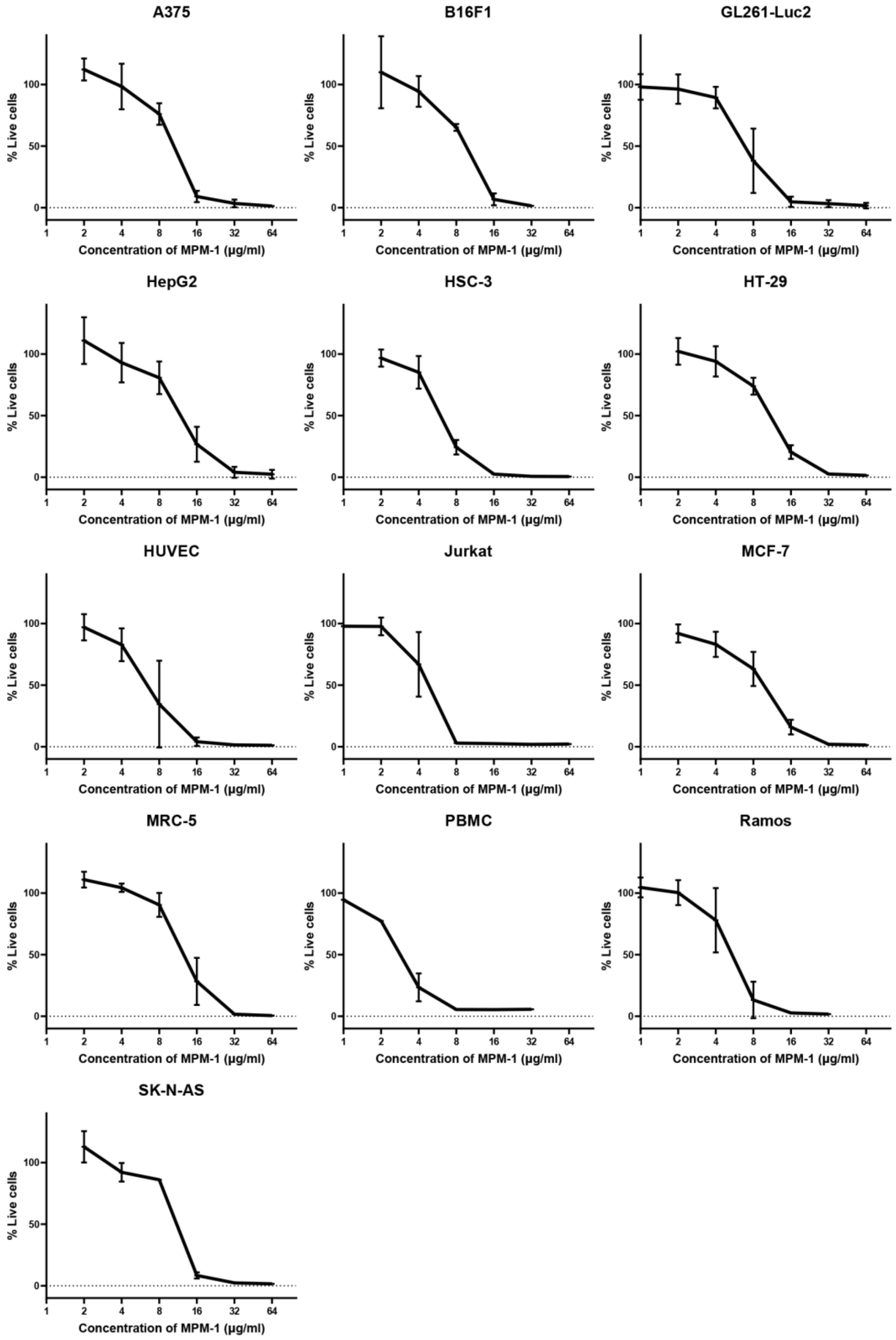

Supplementary Figure S1. Dose-response curves for all cell lines tested against MPM-1. The data is based on three independent MTS experiments, and error bars represent the standard deviation.

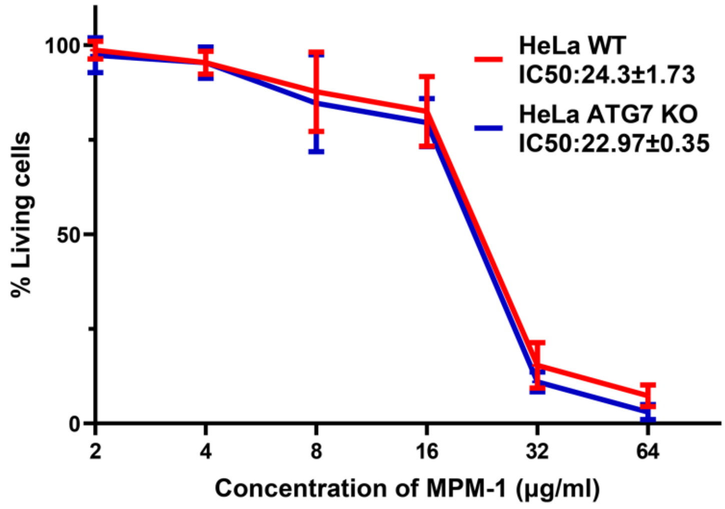

Supplementary Figure S2. Dose-response curves for wild type HeLa cells and ATG7 knockout HeLa cells treated with MPM-1. The data is based on three independent MTS experiments, and error bars represent the standard deviation.

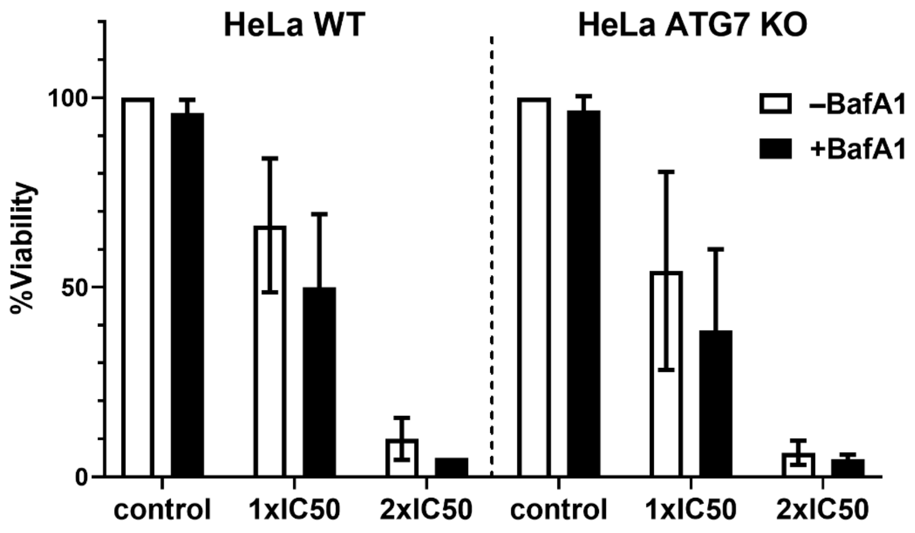

Supplementary Figure S3. Viability of wild type HeLa cells and ATG7 knockout HeLa cells was measured upon treatment with MPM-1 in the presence or absence of Bafilomycin A1 (100 nM). The concentration of MPM-1 was equal to 1xIC50 (23 µg/ml) or 2xIC50 (46 µg/ml). The data is based on three independent MTS experiments, and error bars represent the standard deviation.

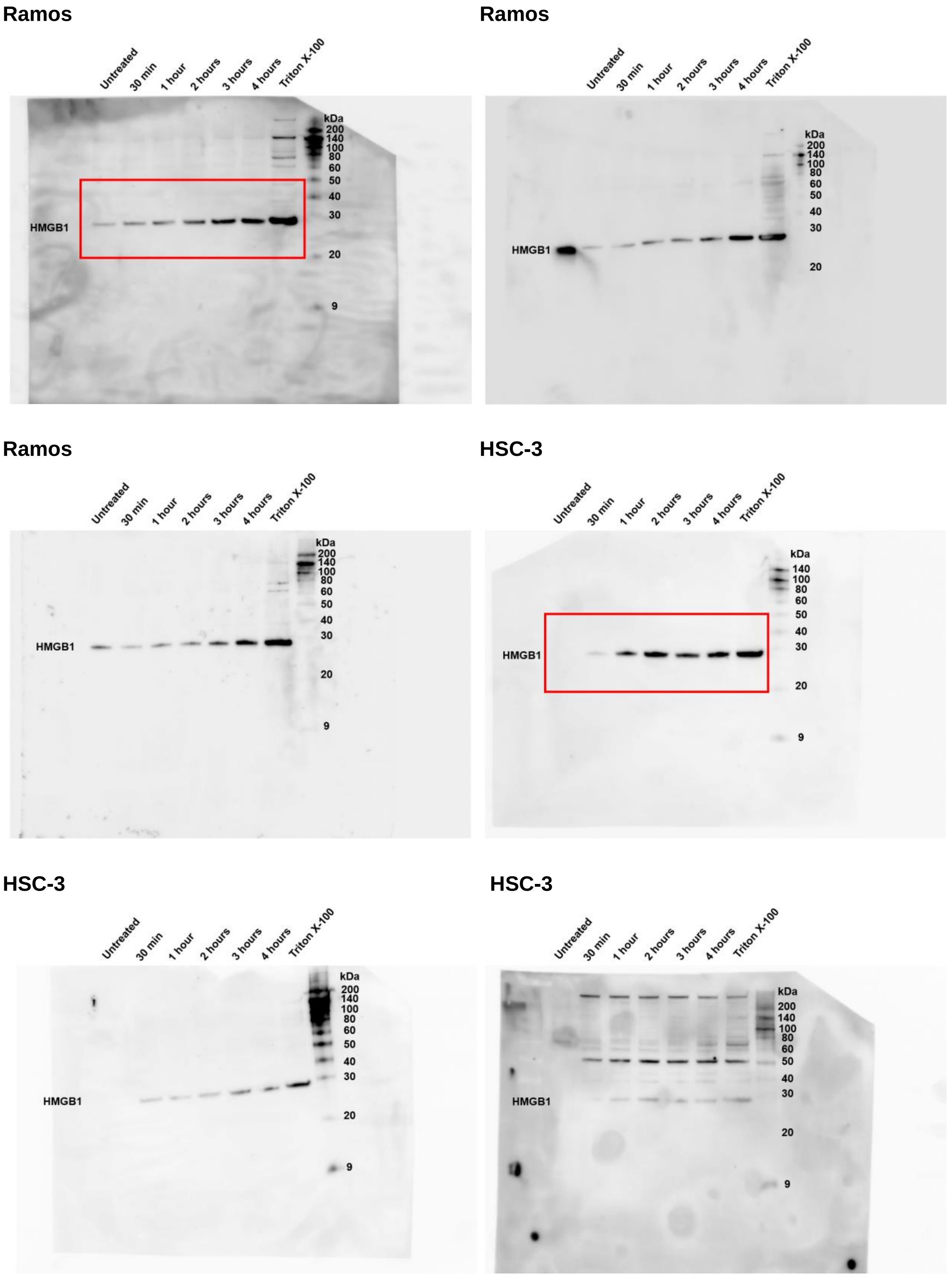

Supplementary Figure S4. Uncropped images of blots presented in Figure 6c in the main article as well as the two additional replicates.

Supplementary Video S1. HSC-3 cells treated with 1xIC50 (8.5 µM) MPM-1.

Supplementary Video S2. HSC-3 cells treated with ½xIC50 (4.25 µM) MPM-1.
